# Effectiveness of Cell-Based Quadrivalent Seasonal Influenza Vaccine: A Systematic Review and Meta-Analysis

**DOI:** 10.3390/vaccines11101607

**Published:** 2023-10-17

**Authors:** Brenda L. Coleman, Iris Gutmanis, Ian McGovern, Mendel Haag

**Affiliations:** 1 Sinai Health, Toronto, ON M5G 2A2, Canada; iris.gutmanis@sinaihealth.ca; 2 Dalla Lana School of Public Health, University of Toronto, Toronto, ON M5T 3M7, Canada; 3 Department of Epidemiology and Biostatistics, Schulich School of Medicine and Dentistry, Western University, London, ON N6A 5C1, Canada; 4 CSL Seqirus, Cambridge, MA 02139, USA; 5 CSL Seqirus, 1105 BJ Amsterdam, The Netherlands

**Keywords:** influenza, quadrivalent influenza vaccine, vaccine effectiveness, cell-based

## Abstract

Cell-based seasonal influenza vaccine viruses may more closely match recommended vaccine strains than egg-based options. We sought to evaluate the effectiveness of seasonal cell-based quadrivalent influenza vaccine (QIVc), as reported in the published literature. A systematic literature review was conducted (PROSPERO CRD42020160851) to identify publications reporting on the effectiveness of QIVc in persons aged ≥6 months relative to no vaccination or to standard-dose, egg-based quadrivalent or trivalent influenza vaccines (QIVe/TIVe). Publications from between 1 January 2016 and 25 February 2022 were considered. The review identified 18 relevant publications spanning three influenza seasons from the 2017–2020 period, with an overall pooled relative vaccine effectiveness (rVE) of 8.4% (95% CI, 6.5–10.2%) for QIVc vs. QIVe/TIVe. Among persons aged 4–64 years, the pooled rVE was 16.2% (95% CI, 7.6–24.8%) for 2017–2018, 6.1% (4.9–7.3%) for 2018–2019, and 10.1% (6.3–14.0%) for 2019–2020. For adults aged ≥65 years, the pooled rVE was 9.9% (95% CI, 6.9–12.9%) in the egg-adapted 2017–2018 season, whereas there was no significant difference in 2018–2019. For persons aged 4–64 years, QIVc was consistently more effective than QIVe/TIVe over the three influenza seasons. For persons aged ≥65 years, protection with QIVc was greater than QIVe or TIVe during the 2017–2018 season and comparable in 2018–2019.

## 1. Introduction

Most licensed influenza vaccines are manufactured using hens’ eggs as the substrate for vaccine virus replication [1]. However, human influenza strains often do not replicate well in eggs, and selective pressure promotes mutations in the antigen-binding sites of egg-grown strains—a process termed “egg adaptation” [2]. Expert consensus estimates that egg adaptation may reduce vaccine effectiveness by as much as 16% [3]. To address these deficiencies, alternatives to hens’ egg–based production have been developed, including cell culture-derived and recombinant vaccines [4,5].

Currently licensed mammalian cell-based influenza vaccines are manufactured using the Madin Darby canine kidney (MDCK) line that was first approved by the US Food and Drug Administration (FDA) in 2012 [6]. MDCK cell-based influenza vaccines include Celtura (Seqirus, Parkville, Australia), a monovalent MF59^®^-adjuvanted MDCK cell vaccine used against the 2009 pandemic strain (A/California/7/2009), as well as a trivalent seasonal inactivated influenza vaccine (TIVc) known as Optaflu^®^ in Europe and Australia and Flucelvax trivalent^®^ in the US (Seqirus, Parkville, Australia), and a quadrivalent seasonal inactivated influenza vaccine (QIVc) known as Flucelvax quadrivalent^®^ in the US and Canada and Flucelvax tetra^®^ in the EU (Seqirus, Parkville, Australia) [7,8]. QIVc was first licensed in the US for individuals ≥ 4 years of age in 2016 and for persons ≥ 6 months of age in 2021 [9]. Initially, the seed strains used in the manufacture of QIVc were derived from eggs due to a lack of available cell-seed strains from the World Health Organization (WHO), which left open the possibility for egg adaptation to occur during the propagation of the candidate vaccine viruses. In 2017–2018, the A(H3N2) seed strain was propagated in MDCK cells, followed by both B strains in 2018–2019 [8,10]. As of the 2019–2020 influenza season, all four strains have been propagated in cell culture for the seed and vaccine virus [11].

Numerous studies have been conducted examining the relative effectiveness of cell-based vs. egg-based influenza vaccines, but the few systematic reviews and meta-analyses that have been conducted have usually focused on efficacy in clinical trials [12,13]. The objective of this study was to evaluate the effectiveness of vaccination with seasonal QIVc relative to vaccination with standard-dose egg-based influenza vaccines or no vaccination among persons ≥ 6 months of age through a systematic review of the literature and a meta-analysis of real-world evidence.

## 2. Materials and Methods

This systematic review and meta-analysis was conducted using the Cochrane methodological standards and the 2015 Preferred Reporting Items for Systematic Reviews and Meta-Analysis Protocols (PRISMA-P) guidelines [14,15]. A protocol outlining the objectives, exclusion criteria, and methods of analysis was specified in advance and registered with the International Prospective Register of Systematic Reviews (PROSPERO) (#CRD42020160851) prior to the start of the search.

### 2.1. Eligibility Criteria

Data from real world evidence studies including cohort, case-control (test-negative), and cluster-randomized studies from the 2016–2017 season onward that were available by 25 February 2022, were eligible for inclusion. Information from full text articles, posters, slide presentations, and abstracts was included if they described, at a minimum, the study design, season(s), region/country, patient population, age groups, how vaccination status was defined, case/outcome definition, adjusted VE and/or rVE and confidence bands, and the variables by which the VE estimates were adjusted. Our review was limited to information published in English, French, Italian, or Spanish and to human studies.

### 2.2. Sources of Information

A professional librarian conducted the literature search in conjunction with the principal investigator. Bibliographic databases including Cochrane, Embase, MEDLINE(R), Medline Epub Ahead of Print, Medline-in-Process, Scopus, Dissertation Abstracts/Proquest, and Web of Science were searched to locate articles indexed as of 25 February 2022. Search terms used with electronic databases included the following: Flucelvax, cell-based, Madin Darby canine kidney cells, MDCK, vaccine, quadrivalent, influenza, influenza A virus, and influenza B virus. The search terms used for each database are detailed in the Supporting Information.

A grey literature search included government sites, sentinel articles, conference abstracts, and meta search engines. Reference lists were hand searched and the principal investigators or their representatives were contacted for additional information.

### 2.3. Study Selection

Two reviewers (B.L.C. and I.G.) independently assessed non-duplicate titles and abstracts to identify potential literature. If only conference titles or abstracts were found, key informants were contacted. For the full document review, the two reviewers independently abstracted data from each eligible reference using pre-defined fields in a data extraction sheet. If more than one source used the same study population, intervention, comparison, and outcome, the more inclusive and complete source was used, with preference given to peer-reviewed publications.

Reviewers came to agreement on the eligibility of research and the data abstracted through consensus. The data quality of all abstracted data elements was assessed by the reviewers.

### 2.4. Exposure of Interest, Study Populations, and Outcomes

The exposure of interest was vaccination with QIVc compared with no vaccination for absolute vaccine effectiveness (aVE) or vaccination with standard dose, egg-based TIV or QIV without an adjuvant for relative vaccine effectiveness (rVE).

Effectiveness estimates were included for persons aged ≥6 months, the lowest licensed age indication of QIVc [16]. For the individual studies, the applied cut-off point was for the applicable licensed age indication in the season and location of the original study.

The primary outcomes of interest were those based on either a clinical diagnosis of influenza or laboratory-confirmed influenza, including a positive test using polymerase chain reaction (PCR; multiplex, real time, or reverse transcription) or culture. Clinical diagnosis of influenza without laboratory confirmation could include a clinical diagnosis by a healthcare provider based on symptoms, as defined by the study protocol or by using diagnostic coding from healthcare databases. Estimates of effectiveness against influenza-related outcomes including pneumonia, hospital admission, intensive care unit admission, or death were also eligible for inclusion. There was no restriction imposed on the clinical setting in which the studies were conducted.

### 2.5. Data Presentation

Absolute VE and rVE against influenza types, subtypes, or lineages were presented separately because the cell-based candidate vaccine viruses used for QIVc production were introduced over several years (A(H3N2) seed strain in 2017–2018 and B seed strains in 2018–2019 [8,10]. Information on potentially confounding factors and the methods used to formulate these adjustments is reported. The results of both primary and secondary analyses of individual reports were tabulated.

### 2.6. Meta-Analyses

Meta-analyses were conducted separately for aVE and rVE estimates. As variation across seasons, country of study, clinical settings, ages, and study designs was expected, the DerSimonian and Laird random effects method was applied using Stata SE v16.1 to conduct meta-analyses [17]. Only adjusted estimates were used to reduce the impact of confounding, and effect sizes were calculated using the adjusted aVE or rVE estimates rather than raw data [18]. Effect estimates could be adjusted hazard ratios (HR), odds ratios (OR), risk ratios (RR), or incidence rate ratios (IRR), depending on the study design. We pooled OR with IRR or RR because the outcomes of interest were likely to meet the rare diseases assumption (outcome occurred in ≤5% of the unexposed population) [19]. Studies reporting HRs were reported and/or analyzed separately.

The I^2^ statistic was used to estimate the total variation across study results that may have been due to heterogeneity rather than chance alone [20]. I^2^ less than 30% is considered low, with increasing heterogeneity as it increases.

Preference was given to results based on the study’s primary analyses to avoid repeated contribution biases. If results for more than one method used to adjust for confounding were available, the one that increased comparability with other studies in that analysis was chosen. Estimates without accompanying confidence intervals were not eligible for use in the meta-analyses since the study weights could not be calculated.

To determine whether the rVE of QIVc vs. QIVe (or QIVe/TIVe) varied by season, clinical outcome, age group, or risk of complications, we conducted a meta-analysis of the available data without regard to other potential sources of heterogeneity (e.g., clinical outcomes were not separated when analyzing seasonal estimates). If studies reported more than one outcome, the more inclusive was selected (e.g., hospital admission or emergency department (ED) visit or outpatient visit took precedence over hospital admission alone). Non-overlapping age groups and risk levels were selected to ensure that representation was as unbiased as possible.

### 2.7. Risk of Bias Assessment

Risk of bias was assessed using the risk of bias in non-randomized studies of interventions (ROBINS-I) tool [21]. This tool guides the reviewer in assessing the risk of bias pertaining to confounding, the selection of participants into the study, the classification of interventions, deviation from intended interventions, missing data, measurement of outcomes, and reported results with an overall judgement about the risk of bias. For the overall risk of bias to be considered low, the study must be equivalent to a well-performed randomized trial [21]. Reviewers discussed any discrepant results to reach consensus, and consensus was always reached.

### 2.8. Deviations from Protocol

The protocol specified that data may be pooled within age groups (e.g., 4–17 years, 18–64 years, ≥65 years) if ≥70% of the population fell within the age group. However, this criterion left only one or two studies for most forest plots. Given the limited number of feasible meta-analyses resulting from this criterion, studies were pooled across different non-overlapping age groups to improve the ability to evaluate the contribution of season and outcome to variability in effect estimates. Outcomes were also pooled by age group where feasible.

## 3. Results

### 3.1. Study Selection

**Figure 1:**
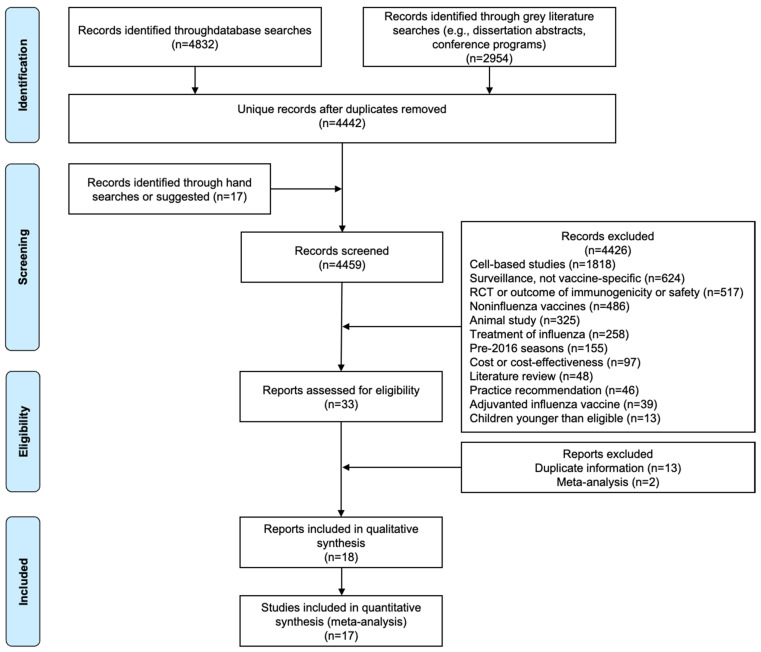
Preferred Reporting Items for Systematic Reviews and Meta-Analysis Protocols (PRISMA-P) flow sheet detailing study selection.

The search criteria yielded 7786 potentially relevant data sources, indexed as of 23–25 February 2022; 4832 through electronic database searches and 2954 through grey literature searches. After removing duplicates, 4442 records were available. Hand searching, examination of review papers, and correspondence with experts resulted in an additional 17 titles being identified and screened for potential eligibility. Following exclusion of 4426 records (see Figure 1 for exclusions), 33 were assessed for eligibility (Figure 1; Table S1), and 15 records were excluded for having data from the same study (e.g., abstracts with subsequent publications) [22,23,24,25,26,27,28,29,30,31,32,33,34,35,36,37,38,39,40,41,42,43,44]. Eighteen records, including four that were identified through hand searching or through peers, contained unique information and were included in the review (Figure 1). All included references were available in English. Results from Klein et al. were not eligible for inclusion in the meta-analyses because they reported hazard ratios and estimates based on the type of influenza rather than all types combined, as in the other studies [22].

### 3.2. Study Characteristics and Risk of Bias Assessment

The 18 studies were conducted during the 2017–2018 (n = 8), 2018–2019 (n = 5), and 2019–2020 (n = 5) seasons in the UK (n = 1) and US (n = 17); five evaluated aVE based on laboratory-confirmed influenza, and 17 assessed rVE based on laboratory-confirmed influenza (n = 6) or clinical data (n = 11) (Table 1). The overall study populations included in the analyses comprised subjects aged 4–17 years (n = 1), ≥4 years (n = 5), ≥18 years (n = 5), 4–64 years (n = 4), and ≥65 years (n = 3). One study included persons aged ≥6 months to <4 years [23], but as noted below, this age group was not included because of a critical risk of bias in the analysis, since the vaccine was not licensed in this age group during the seasons included. Seven studies were a test-negative design, of which two also had cohort design elements [22,23,24,25,26,27,28], whereas the other 11 were strictly cohort designs that used data gleaned from electronic medical records and claims data (Table 1) [29,30,31,32,33,34,35,36,37,38,39]. Six of the seven test-negative design studies reported adjusted aVE of QIVc and QIVe or QIVe/TIVe. Three of the six studies that examined hospital encounters (either an admission or ED visit) used code set B (International Classification of Diseases, 10th Edition, (ICD10) J9-J11) plus J129 (viral pneumonia, unspecified) [37,38,39]. The other three used code set B to identify influenza-related claims [32,33,34]. The rVE of QIVc compared with QIVe or QIVe/TIVe was available for 11 of 18 studies in which it was compared with QIVe/TIVe in six records (Table S2). Two studies [35,36] that were only available as congress abstracts at the time of the meta-analysis have since been published in the peer-reviewed literature [45,46].

In six reports, multivariable regression was used to adjust for potential confounders [23,24,25,26,28]. Boikos et al. used propensity scores to adjust for potential confounders in their 2020 publication [31], whereas propensity scores with inverse probability of treatment weights (IPTW) were used to adjust for imbalances between vaccine recipient groups in three reports by Divino and Krishnarajah [32,33,34]. In another four reports, both IPTW and doubly robust IPTW results were reported [29,37,38,39]. In two abstracts/posters originating from one study, only the doubly robust IPTW results were reported (Table S2) [35,36].

The overall risk of bias was moderate for 17 of 18 studies included in the qualitative synthesis, based on the ROBINS-I tool (Table S3). The annual surveillance report by Public Health England was assessed as having a serious risk of bias because the source of the data contained very limited methodological information accompanying the results. Specifically, the report lacked details necessary to assess the risk of confounding, selection bias, missing data, or misclassification of outcomes [27]. In addition, in one their analyses, DeMarcus et al. included children younger than 4 years, the licensed age indication for QIVc in the US during the 2017–2018 season in which the study was conducted [23]. As such, the results for this age group were not included in this review.

We did not detect evidence of publication/reporting bias for the 17 reports used in the meta-analyses (Egger *p*-value = 0.62).

### 3.3. Absolute Vaccine Effectiveness of QIVc

Five studies reported the adjusted aVE of QIVc against laboratory-confirmed influenza (Figure S1) [23,24,25,27,28]. The pooled aVE estimate for QIVc was 37.6% (95% CI, 19.4% to 55.9%; I^2^ = 45%), including estimates from 2017–2018 through 2019–2020. All original estimates were adjusted for various potential confounders, and the pooled estimate had moderate heterogeneity. Further stratification was not feasible due to the paucity of data for aVE using QIVc. The pooled estimate for TIVe/QIVe from the same studies was 26.1% (95% CI, 6.7% to 45.4%, I^2^ = 81%).

The report by Public Health England [27] was assessed as having a serious risk of bias. As such, a sensitivity analysis was conducting excluding these estimates. The resulting pooled aVE estimate was lower for both types of vaccine at 30.9% (95% CI, 9.4% to 52.3%; I^2^ = 42%) for QIVc and 20.8% (95% CI, 1.6% to 40.1%; I^2^ = 80%) for TIVe/QIVe.

### 3.4. Relative Vaccine Effectiveness of QIVc Compared with QIVe/TIVe

The overall pooled rVE of QIVc vs. QIVe/TIVe was estimated as 8.4% (95% CI, 6.5% to 10.2%) across all seasons, ages, and study designs (Figure S2a). However, heterogeneity was moderate to substantial at 56%. Figure S2b presents a post-hoc Galbraith plot for the studies included in these analyses. All results were within the 95% confidence band. Subgroup analyses were completed to determine sources of variation in sources of heterogeneity.

#### 3.4.1. Comparisons by Study Design

As shown in Figure S2a, test-negative studies in which patients were tested for influenza had a pooled rVE of 5.0% (95% CI, −6.0% to 16.0%; I^2^ = 0) [23,24,25,26,28]. For cohort design studies that depended on diagnostic coding for influenza-related illnesses, rVE was 8.5% (95% CI, 6.5% to10.4%; I^2^ = 70%) [29,31,32,33,34,35,36,37,38,39]. The confidence intervals for the cohort studies were narrower than those for the test-negative studies. The confidence intervals for the cohort studies were narrower than those for the test-negative studies, possibly leading to higher estimates of heterogeneity.

#### 3.4.2. Laboratory-Confirmed Influenza

In the four studies using laboratory-confirmed outcomes during the 2017–2018 and 2018–2019 seasons, the rVE of QIVc was similar to that of QIVe/TIVe for any type of influenza, with a pooled rVE of 5% (95% CI, −6.0% to 16.0%; I^2^ = 0) across the two seasons (Figure S3a) [22,23,24,25]. Relative VE data by influenza type were available for influenza A (any influenza A or A(H3N2)) from the 2017–2018 season and 2018–2019 season, but not for the 2019–2020 season. The rVE of QIVc versus QIVe/TIVe against any influenza A was 9.5% (95% CI, −3.8% to 22.9%) (Figure S3b). The rVE of QIVc vs. QIVe/TIVe against influenza A(H3N2) specifically was 21.1% (95% CI, 1.6% to 40.5%), driven by the studies conducted in the 2017–2018 season, during which this strain was dominant (Figure S3c) [23,24,25,28]. Restricting data to the 2017–2018 season alone, the rVEs of influenza A and influenza A(H3N2) were 13.3% (95% CI, −1.1% to 27.8%; I^2^ = 0) and 25.3% (95% CI, 4.7% to 46.0%; I^2^ = 0), respectively.

In the 2017–2018 influenza season, the TIV formulations included the Victoria lineage, while the Yamagata lineage was the predominant circulating B strain, detected in about 20% of cases in in the US [47]. Since the minimum criteria of three studies comparing against QIVe only was not met, we were unable to pool estimates of rVE against influenza B [22,23,24,28].

#### 3.4.3. Comparisons by Clinical Setting

The included studies evaluated rVE in a number of clinical settings including outpatient visits, ED visits, and inpatient hospitalizations, as well as the combination of all three. Meta-analyses were conducted stratified by clinical setting to evaluate the impact of the setting on the pooled estimate. The pooled rVE estimate for prevention of an influenza-related medical encounter in any clinical setting (combination of outpatient visit, ED visit, or hospital admission) was 9.0% (95% CI, 7.0% to 11.0%; I^2^ = 70%), with three cohort design studies contributing to the estimate (Figure S4) [26,29,35,36]. The pooled rVE estimate for prevention of influenza-related outpatient visits was 11.4% (95% CI, 9.7% to 13.0%; I^2^ = 0), with four of the five studies conducted in the 2017–2018 season (Figure S4) [23,26,31,36,37].

Prevention of influenza ED visits or hospital admissions was reported in six studies, with a pooled rVE estimate of 7.5% (95% CI, 5.6% to 9.3%; I^2^ = 71%) (Figure S4) [32,33,34,37,38,39]. Prevention of hospital admissions was reported in 11 studies, with a pooled rVE estimate of 10.7% (95% CI, 10.0% to 11.4%; I^2^ = 87%) (Figure S4) [24,25,26,28,32,33,34,36,37,38,39].

#### 3.4.4. Comparisons by Season

Across the three seasons, the pooled rVE of QIVc vs. QIVe/TIVe was 11.4% (95% CI, 9.0% to 13.9%) in 2017–2018, 6.0% (95% CI, 2.8% to 9.1%) in 2018–2019, and 7.7% (95% CI, 4.2% to 11.3%) in 2019–2020 (Figure S5). These pooled rVE estimates were based on all ages, settings, and designs (test negative and cohort).

#### 3.4.5. Comparisons by Age Group

The pooled rVE estimates of QIVc vs. QIVe/TIVe were 8.0% (95% CI, 1.6% to 14.4%) for children and adolescents aged 4–17 years, 9.9% (95% CI, 1.4% to 18.4%) for persons aged 4–64 years, 9.9% (95% CI, 5.3% to 14.5%) for adults aged 18–64 years, and 0.5% (95% CI, −5.7% to 6.8%) for adults aged ≥65 years (Figure S6). Data in each age group originated from three different influenza seasons. Estimates of heterogeneity were substantial to considerable for each age group.

#### 3.4.6. Comparisons by Age Group and Season

**Figure 2:**
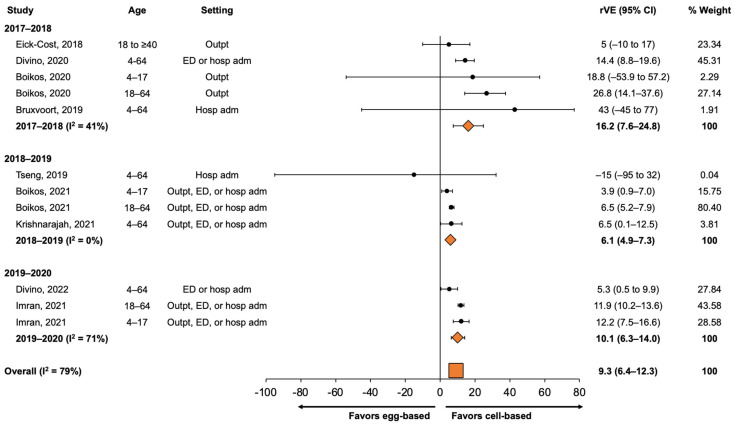
Forest plot of rVE estimates of QIVc compared with QIVe (or QIVe/TIVe) in preventing medical encounters for persons aged 4–64 years, 2017–2018 to 2019–2020. Pooled rVE estimates were calculated separately for each season. adm, admission; CI, confidence interval; ED, emergency department; hosp; hospital; I sq, I^2^ (heterogeneity measure); Outpt, outpatient; QIVc, cell-based quadrivalent inactivated influenza vaccine; QIVe, egg-based quadrivalent inactivated influenza vaccine; rVE, relative vaccine effectiveness; TIVe, egg-based trivalent influenza vaccine [24,25,26,29,30,31,32,33,34,35,36].

Within different subgroup analyses, age (4–64 years and ≥65 years of age) and season had the greatest variability in pooled estimates. To further assess the heterogeneity, pooled estimates by season were determined for populations 4–64 years and ≥65 years of age. For persons aged 4–64 years (Figure 2), the rVE for QIVc vs. QIVe/TIVe was 16.2% (95% CI, 7.6% to 24.8%) in 2017–2018, 6.1% (95% CI, 4.9% to 7.3%) in 2018–2019, and 10.1% (95% CI, 6.3% to 14.0%) in 2019–2020, with an overall pooled estimate of 9.3% (95% CI, 6.4% to 12.3%). Considerable heterogeneity remained, with an I^2^ of 79%.

**Figure 3:**
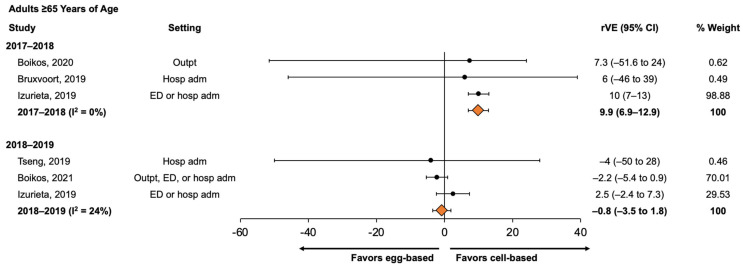
Forest plot of rVE estimates of QIVc compared with QIVe (or QIVe/TIVe) in preventing medical encounters for adults ≥65 years of age, by season, 2017–2018 and 2018–2019. Pooled rVE estimates were calculated separately for each season. adm, admission; ED, emergency department; Hosp, hospital; I sq, I^2^ (heterogeneity measure); Outpt, outpatient; QIVc, cell-based quadrivalent inactivated influenza vaccine; QIVe, egg-based quadrivalent inactivated influenza vaccine; rVE, relative vaccine effectiveness; TIVe, egg-based trivalent inactivated influenza vaccine [24,25,29,30,31,37].

For adults 65 years of age or older, QIVc was significantly more effective than QIVe/TIVe in the 2017–2018 influenza season (rVE, 9.9% (95% CI, 6.9% to 12.9%)), whereas the pooled rVE estimate for 2018–2019 was −0.8% (95% CI, −3.5% to 1.8%), indicating no significant difference in the effectiveness of QIVc and QIVe/TIVe (Figure 3).

#### 3.4.7. Comparisons in Persons at Risk of Serious Influenza Complications

Finally, we evaluated how vaccine effectiveness compared for persons at high risk of serious influenza complications compared to the overall population. Four studies compared the effectiveness of QIVc to QIVe for both the general population and populations at higher risk of serious outcomes due to influenza. Boikos et al. defined higher risk populations as having one or more of the following: chronic pulmonary disease, asthma, myocardial infarction, congestive heart failure, cerebrovascular disease, peripheral vascular disease, renal disease, diabetes, any malignancy and/or metastatic solid tumors, HIV/AIDS, rheumatic disease, or liver disease [30], whereas the papers by Divino et al. and Krishnarajah et al. defined higher risk populations as having asplenia or dysfunction of the spleen, chronic heart disease, chronic kidney disease, chronic liver disease, chronic neurological disease, chronic respiratory disease, diabetes, immunosuppression, morbid obesity, or pregnancy [32,33,34]. The pooled rVE estimate for QIVc vs. QIVe for higher risk populations was 10.3% (95% CI, 5.7% to 15.0%; I^2^ = 52%), whereas the rVE in the general population was 8.2% (95% CI, 5.1% to 11.3%; I^2^ = 58%) (Figure S7). These results demonstrate that a benefit is maintained in the higher risk population, which is more at risk of having severe complications due to an influenza infection.

## 4. Discussion

This systematic literature review using real world evidence of the effectiveness of QIVc spans three influenza seasons (2017–2018 through 2019–2020), from the first use of QIVc with a candidate vaccine virus that was derived and propagated in mammalian cell culture for the influenza A(H3N2) strain through to the introduction of the cell cultured strain for all subtypes of influenza in 2019–2020 and the beginning of the coronavirus disease 2019 (COVID-19) pandemic. Of 4459 unique records screened, 18 met the inclusion criteria in this review. Compared with standard dose, egg-based influenza vaccines, QIVc was significantly more effective at reducing medical encounters, with an overall pooled rVE of 8.4% (95% CI, 6.5% to 10.2%) vs. QIVe/TIVe when pooled across different seasons, outcomes, and age groups. Based on an assessment of bias with the ROBINS-I tool, this systematic review and meta-analyses provides moderately strong evidence that QIVc is more protective against any medical encounter related to influenza compared with QIVe/TIVe for people 4–64 years of age.

Irrespective of age groups and outcomes, the greatest benefit of QIVc relative to egg-based vaccines was seen in the 2017–2018 season (pooled estimate 11.4% (95% CI, 9.0% to 13.9%)). Significant benefits were also observed in the other two seasons, albeit at a lesser magnitude (2018–2019: 6.0% (95% CI, 2.8% to 9.1%); 2019–2020: 7.7% (95% CI, 4.2% to 11.3%)). The effect during the 2017–2018 season may have been related to documented egg-adaptation that occurred in the influenza A(H3N2) strain during production of egg-based vaccines, which does not affect QIVc [48]. This was supported by a season and strain specific analysis for the 2017–2018 season showing an rVE for influenza A(H3N2) of 25.3% (95% CI, 4.7% to 46.0%; I^2^ = 0). In the US 2017–2018 season, egg adaptation in the A(H3N2) vaccine virus was considered a major cause of antigenic mismatch and reduced vaccine effectiveness in this season, with the egg-based candidate vaccine virus (CVV) (A/Hong Kong/4801/2014) having acquired the T160K, L94P, and N96S egg adaptive mutations, which have been shown to alter the antigenicity of the vaccine [2,49,50]. The US 2018–2019 season initially had predominant circulation of A(H1N1) followed by co-circulation with A(H3N2) viruses that drifted away from the vaccine strain towards the end of the season [51]. Both egg-adaptation and drift contributed to a low observed overall vaccine effectiveness [49]. In this season, the CVV for A(H1N1) of the QIVc vaccine was not yet cell-derived and thus could not contribute to the benefit for the QIVc vaccine. Finally, the 2019–2020 season was characterized by pre-dominant circulation of B/Victoria and A(H1N1) viruses [52]. For the first time, all CVVs for QIVc were cell-derived. Antigenic characterization suggested that egg-adaptation occurred with the B/Victoria lineage [53]. Assays from human serology indicated that circulating A(H1N1) viruses were affected by potential egg adaptation in combination with drift [54].

Our analyses showed that QIVc was significantly more effective at preventing medical encounters than QIVe in adults ≥65 years of age during the 2017–2018 season, which was not observed in the 2018–2019 season. In contrast, QIVc was consistently more effective than QIVe/TIVe for people 4–64 years of age across all three seasons. With the increasing recognition of the challenges of the declining immune response to infection and immunization with older age, vaccines with adjuvant or higher antigen dose have become more commonly preferred and also preferentially recommended for the ≥65 year age group. Given that the vaccines of interest concern both non-adjuvanted standard vaccines, it may be that for the ≥65 year age group, the advantage of cell-based vaccine in avoiding egg adapted mutations is of greater clinical significance in seasons with larger differences between cell-based and egg-based vaccine effectiveness.

In 1995, the WHO recommended the development of alternatives to egg-based influenza vaccines due to the potential for egg adaptation, as well as possible disruptions in the supply of eggs, which could reduce vaccine supplies [1,4]. Since that time, as cell-based and recombinant influenza vaccines have become available, numerous studies have shown the benefits of these approaches over egg-based vaccines. Mannocci and colleagues found that immunogenicity results favored cell-based and recombinant influenza vaccines over standard-dose egg-based vaccines for the A(H3N2) and B influenza strains in a meta-analysis of 24 randomized, controlled trials in adults 18–64 years of age [12]. In a systematic review of 19 randomized, controlled trials and observational studies of cell-based influenza vaccines, Jordan and colleagues found that cell-based influenza vaccines were safe and effective and prevented significantly more influenza-related hospitalizations, hospital encounters, and outpatient visits than egg-based vaccines, although these authors also reported that a small amount of heterogeneous data limited their conclusions, similar to our findings [13]. Puig-Barbera and colleagues conducted a similar review of QIVc effectiveness among adults 18 years and older [55]. The authors calculated a pooled a rVE for the 2017–2018 influenza season of 11% (8% to 14%) favoring QIVc but did not find a benefit of QIVc for the 2018–2019 season. There was strong evidence supporting age as a significant driver of the heterogeneity. When the analysis was restricted to subjects 18–64 years of age, the benefit of QIVc was also observed for the 2018–2019 season, although it was smaller in magnitude than for the 2017–2018 season. These findings align with the findings from the current review.

This review and meta-analysis had several strengths. It followed the PRISMA-P guidelines, used the ROBINS-I tool to assess sources of bias, performed meta-analyses using a random effects approach (in anticipation of true variation in vaccine effectiveness across different populations and studies), and used only the adjusted estimates provided by the authors. Also, data on absolute and relative vaccine effectiveness were available for multiple seasons. Like all systematic reviews, however, this study also had several limitations. Several of the data sources available at the time of the review, such as scientific conference posters and presentations, were not peer-reviewed and lacked the depth of detail preferred for systematic reviews and meta-analyses. Data sources included both test-negative and cohort designs that used various statistical methods to adjust for confounding (regression modelling, propensity score modelling, IPTW, DR-IPTW) and to report the results (e.g., IRR, OR, HR). Opportunities to estimate type-specific vaccine effectiveness are few, given the considerable size of the (underlying) cohort needed, particularly for relative vaccine effectiveness. Therefore, the availability of 18 studies over a three season period is considerable. However, many factors related to host, virus, and vaccine affect vaccine effectiveness, and estimates can vary by study design and setting. Hence, despite the substantial number of studies, the paucity of rVE data regarding the drivers of vaccine effectiveness nevertheless meant that we needed to combine various ages, settings, seasons, and adjustment techniques. As such, the heterogeneity between studies was often substantial, which limited the certainty of some of the pooled estimates provided in this review.

## 5. Conclusions

Our systematic review and meta-analyses of real-world evidence provides additional support that QIVc provides greater protection against any influenza-related medical encounters relative to QIVe/TIVe for people 4–64 years of age. The benefit of QIVc for people 4–64 years of age was particularly robust during the 2017–2018 season, when the effectiveness of egg-based influenza vaccines was reduced by egg adaptation in the influenza A(H3N2) strain. For persons aged ≥65 years, the benefits of QIVc were inconsistent; protection with QIVc was greater than QIVe or TIVe during the 2017–2018 season but comparable in 2018–2019. Given the variability of seasonal characteristics, the generation of further evidence on the effectiveness of cell-based vaccines relative to egg-based vaccines is of interest to further understand how variations in seasonal characteristics contribute to the observed results. Additionally, future research studies may be able to evaluate the effectiveness of QIVc among children 6 months to <4 years of age due to the more recently expanded age indication of QIVc [9].

## Figures and Tables

**Table 1:** Characteristics of included studies.

				QIVc Compared with:		
First Author, Year	Country	Season	Study Design	No Vaccine	TIVe/QIVe	QIVe
Boikos, 2020 [31]	US	2017–2018	Cohort			X
Boikos, 2021 [30]	US	2018–2019	Cohort			X
Boikos, 2021 [29]	US	2018–2019	Cohort			X
Bruxvoort, 2019 [24]	US	2017–2018	TND	X	X	
DeMarcus, 2019 [23]	US	2017–2018	TND	X	X	
Divino, 2020 [32]	US	2017–2018	Cohort			X
Divino, 2022 [33]	US	2019–2020	Cohort			X
Eick-Cost, 2018 [26]	US	2017–2018	TND and Cohort		X	
Imran, 2021 [35]	US	2019–2020	Cohort			X
Imran, 2021 [36]	US	2019–2020	Cohort			X
Izurieta, 2019 [37]	US	2017–2018	Cohort		X	X
Izurieta, 2020 [38]	US	2018–2019	Cohort			X
Izurieta, 2021 [39]	US	2019–2020	Cohort			X
Klein, 2020 [22]	US	2017–2018	TND and Cohort	X	X ^a^	
Krishnarajah, 2021 [34]	US	2018–2019	Cohort			X
Martin, 2021 [28]	US	2017–2018	TND	X	X ^b^	
Public Health England, 2020 [27]	UK	2019–2020	TND	X		
Tseng, 2019 [25]	US	2018–2019	TND	X	X	

Abbreviations: QIVc, cell-based quadrivalent inactivated influenza vaccine; QIVe, egg-based quadrivalent inactivated influenza vaccine; TIVe, egg-based, trivalent inactivated influenza vaccine; TND, test negative design. ^a^ TIVe only for influenza B. ^b^ Includes high-dose TIVe and possibly adjuvanted TIVe among those ≥65 years.

## Data Availability

Data available upon request from the authors.

## Supplementary Materials

The following supporting information can be downloaded at https://www.mdpi.com/article/10.3390/vaccines11101607/s1: Table S1: Studies assessed for eligibility; Table S2. Summary of the available aVE and/or rVE data for QIVc, by season, age, influenza definition, vaccine comparator, and population; Table S3: Assessment of risk of bias using ROBINS-I tool for relative vaccine effectiveness estimates (N = 18); Figure S1. Forest plot of aVE estimates of QIVc against laboratory-confirmed influenza (any type), 2017–2018 to 2019–2020; Figure S2: rVE estimates of QIVc compared with QIVe (or QIVe/TIVe) in preventing influenza-related medical encounters, 2017–2018 to 2019–2020; Figure S3: Forest plot of rVE estimates of QIVc compared with QIVe (or QIVe/TIVe) against laboratory-confirmed influenza, by influenza type; Figure S4: Forest plot of rVE estimates of QIVc compared with QIVe (or QIVe/TIVe) in preventing medical encounters by clinical setting, all ages, 2017–2018 to 2019–2020; Figure S5: Forest plot of rVE estimates of QIVc compared with QIVe (or QIVe/TIVe) in preventing medical encounters, by season, 2017–2018 to 2019–2020; Figure S6: Forest plot of rVE estimates of QIVc compared with QIVe (or QIVe/TIVe) in preventing medical encounters by age group, 2017–2018 to 2019–2020; Figure S7: Forest plot of rVE estimates of QIVc compared with QIVe in preventing medical encounters for populations at higher risk of serious outcomes compared with the general population, 2017–2018 to 2019–2020.
