# Supplementary material for: Effectiveness of Cell-Based Quadrivalent Seasonal Influenza Vaccine: A Systematic Review and Meta-Analysis
Source: Vaccines (Basel). 2023 Oct 17;11(10):1607. doi: 10.3390/vaccines11101607 (PMC10610589; doi:10.3390/vaccines11101607)
Supplement: Supplementary file 1 [file vaccines-11-01607-s001.zip › vaccines-2601877-supplementary.pdf]

SUPPORTING INFORMATION FOR:

Vaccine Effectiveness of Cell-Based Quadrivalent Influenza Vaccine: a Systematic Review and Meta-analysis

Brenda Coleman, Iris Gutmanis, Ian McGovern, Mendel Haag

Table of Contents

SEARCH TERMS AND DATABASES 2

TABLE S1. Studies assessed for eligibility 13

TABLE S2. Summary of the available aVE and/or rVE data for QIVc, by season, age, influenza definition, vaccine comparator, and population 15

TABLE S3. Assessment of risk of bias using ROBINS-I tool for relative vaccine effectiveness estimates (N=18) 20

FIGURE S1. Forest plot of aVE estimates of QIVc and QIVe/TIVe against laboratory-confirmed influenza (any type), 2017–2018 to 2019–2020. 22

FIGURE S2. rVE estimates of QIVc compared with QIVe (or QIVe/TIVe) in preventing influenza-related medical encounters, 2017–2018 to 2019–2020. 23

FIGURE S3. Forest plot of rVE estimates of QIVc compared with QIVe (or QIVe/TIVe) against laboratory-confirmed influenza, by influenza type. 24

FIGURE S4. Forest plot of rVE estimates of QIVc compared with QIVe (or QIVe/TIVe) in preventing medical encounters by clinical setting, all ages, 2017–2018 to 2019–2020. 25

FIGURE S5. Forest plot of rVE estimates of QIVc compared with QIVe (or QIVe/TIVe) in preventing medical encounters, by season, 2017–2018 to 2019–2020. 26

FIGURE S6. Forest plot of rVE estimates of QIVc compared with QIVe (or QIVe/TIVe) in preventing medical encounters by age group, 2017–2018 to 2019–2020. 27

FIGURE S7. Forest plot of rVE estimates of QIVc compared with QIVe in preventing medical encounters for populations at higher risk of serious outcomes compared with the general population, 2017–2018 to 2019–2020. 28

References 29

SEARCH TERMS AND DATABASES
Searches were run in the following databases: OvidSP MEDLINE, Medline in Process, Medline Epub Ahead of Print and EMBASE; Cochrane Wiley; Dissertation Abstracts (ProQuest); Clarivate Analytics Web of Science; and Elsevier Scopus; Microsoft Academic Index, Science.gov; conference websites; and governmental websites to include articles indexed as of February 23 to 25, 2022.

A total of 7,786 references were retrieved. All references were saved in an EndNote library that was then used to identify the 3,334 duplicates. There were 4,452 unique references. Hand searches identified an additional 17 references.

Database	Dates	Total	Unique	Duplicates
Cochrane	Issue 2 of 12, February 2022	440	275	165
Embase	1980 to 2022 Week 07	1420	603	817
Dissertation abstracts	February 23, 2022	447	447	0
Hand searches		0	0	0
MEDLINE(R)	1946 to February Week 2 2022	1498	1494	4
Medline Epub Ahead of Print	February 21, 2022	20	20	0
Medline -in-Process	1946 to February 21, 2022	24	24	0
Scopus	to February 21, 2022	619	350	269
Web of Science	to February 21, 2022	811	158	653
18th International Congress on Infectious Diseases	February 24, 2022	61	61	0
AMMI Canada	February 24, 2022	6	6	0
Canadian Immunization Conference	February 24, 2022	17	9	8
ECCMID	February 24, 2022	29	27	2
European Union Publications	February 24, 2022	9	8	1
European Centre for Disease Prevention & Control	February 24, 2022	0	0	0
ICAR	February 24, 2022	1	1	0
ID Week	February 24, 2022	3	2	1
International Journal of Infectious Diseases	February 24, 2022	105	67	38
Options X	February 24, 2022	62	58	4
Google Scholar	February 24, 2022	357	118	239
Microsoft Academic Search	February 24, 2022	1141	436	705
Open Grey	February 24, 2022	0	0	0
Science.gov	February 24, 2022	714	276	438
Health Canada	February 24, 2022	2	2	0
Total		7786	4442	3344

The following tables record the search strategies and the terms used in each of the databases.

MEDLINE

#	Searches	Results	Comment
1	(celtura or "m8-59" or "M8 59").mp.	4	
2	flucelvax.mp.	9	
3	(quadrivalent adj5 (influen* or flu) adj5 vaccin*).mp.	382	
4	qivc.mp.	10	
5	iivc.mp.	14	
6	Optaflu.mp.	13	
7	("cell-based" adj5 (influen* or flu) adj5 vaccin*).mp.	51	
8	(cell adj5 based adj5 influen* adj5 vaccin*).mp.	97	
9	("Cell-Culture*" adj5 (Influenz* or flu) adj5 vaccin*).mp.	145	
10	(Cell adj5 Culture* adj5 (Influenz* or flu) adj5 vaccin*).mp.	146	
11	("cell-derived" adj5 vaccine*).mp.	160	
12	(cell adj2 derived adj5 vaccine*).mp.	370	
13	or/1-12	890	Base clinical set 1 - Cell-based influenza vaccine terms
14	Madin Darby Canine Kidney Cells/	4319	
15	(mdck or (Madin adj2 Darby)).mp.	12047	
16	(mammalian adj5 cell*).mp.	74361	
17	or/14-16	85931	MDCK or mammalian cell terms
18	Influenza Vaccines/	25104	
19	Influenza, Human/	54018	
20	influenza a virus/ or influenza a virus, h1n1 subtype/ or influenza a virus, h3n2 subtype/	39263	
21	influenzavirus b/ or influenza b virus/	4658	
22	influenz*.mp.	133395	
23	"B/Yamagata lineage".mp.	77	
24	"B/Victoria lineage".mp.	97	
25	or/18-24	133395	Influenza/vaccine terms
26	17 and 25	3807	Base clinical set 2 - MDCK and vaccine terms results
27	13 or 26	4577	Base clinical set 2 -mammalian cell based vaccine results
28	limit 27 to human	2878	Human limit
29	(human* or people or man or men or woman or women or male* or female*).mp.	22696051	Human textword terms

30	27 and 29	3413	Human textword results
31	28 or 30	3413	Final results
32	limit 31 to yr="2016 -Current"	1527	Final results publication year limit
33	limit 32 to (english or french or italian or spanish)	1498	Final results language limit

MEDLINE-in-Process & In-Data-Review Citations

#	Searches	Results		Comment
1	(celtura or "m8-59" or "M8 59").mp.	0	0	
2	flucelvax.mp.	3	3	
3	(quadrivalent adj5 (influen* or flu) adj5 vaccin*).mp.	71	44	
4	qivc.mp.	0	3	
5	("cell-based" adj5 (influen* or flu) adj5 vaccin*).mp.	8	7	
6	(cell adj5 based adj5 influen* adj5 vaccin*).mp.	14	7	
7	("Cell-Culture*" adj5 (Influenz* or flu) adj5 vaccin*).mp.	12	4	
8	(Cell adj5 Culture* adj5 (Influenz* or flu) adj5 vaccin*).mp.	12	4	
9	or/1-8	91	50	Base clinical set 1 - Cell-based influenza vaccine terms
10	(mdck or (Madin adj2 Darby)).mp.	466	123	
11	(mammalian adj5 cell*).mp.	4441	1398	
12	or/10-11	4894	1519	MDCK or mammalian cell terms
13	influenz*.mp.	8855	2374	
14	"B/Yamagata lineage".mp.	10	1	
15	"B/Victoria lineage".mp.	7	1	
16	or/13-15	8855	2374	Influenza terms
17	12 and 16	165	49	Base clinical set 2 - MDCK/mammalian cell influenza vaccine results
18	9 or 17	249	97	QIVc or MDCK results
19	(human* or people or man or men or woman or women or male* or female*).mp.	562823	162534	Human textwords
20	18 and 19	74	31	Human limit
21	limit 20 to yr="2016 -Current"	61	31	Final results - Publication year limit

MEDLINE(R) Epub Ahead of Print

#	Searches	Results	Comment
1	(celtura or "m8-59" or "M8 59").mp.	0	
2	flucelvax.mp.	1	
3	(quadrivalent adj5 (influen* or flu) adj5 vaccin*).mp.	12	
4	qivc.mp.	0	
5	iivc.mp.	0	
6	Optaflu.mp.	1	
7	("cell-based" adj5 (influen* or flu) adj5 vaccin*).mp.	1	
8	(cell adj5 based adj5 influen* adj5 vaccin*).mp.	1	
9	("Cell-Culture*" adj5 (Influenz* or flu) adj5 vaccin*).mp.	0	
10	(Cell adj5 Culture* adj5 (Influenz* or flu) adj5 vaccin*).mp.	0	
11	or/1-10 [****Base clinical set 1 - Cell-based influenza vaccine terms****]	13	Base clinical set 1 - Cell-based influenza vaccine terms
12	(mdck or (Madin adj2 Darby)).mp.	29	
13	(mammalian adj5 cell*).mp.	408	
14	or/12-13 [****MDCK or mammalian cell terms****]	435	MDCK or mammalian cell terms
15	influenz*.mp.	1219	
16	"B/Yamagata lineage".mp.	1	
17	"B/Victoria lineage".mp.	0	
18	or/15-17 [****Influenza terms****]	1219	Influenza terms
19	14 and 18 [****Base clinical set 2 - MDCK/mammalian cell influenza vaccine results****]	10	Base clinical set 2 - MDCK/mammalian cell influenza vaccine results
20	11 or 19 [****QIVc or MDCK results****]	23	QIVc or MDCK results
21	limit 20 to yr="2016 -Current"	20	Final results - Publication year limit
22	limit 21 to (english or french or italian or spanish)	20	Final results - language limit

EMBASE

#	Searches	Results	Comment
1	(celtura or "m8-59" or "M8 59").mp.	50	
2	flucelvax.mp.	118	
3	(quadrivalent adj5 influen* adj5 vaccin*).mp.	579	
4	qivc.mp.	20	
5	iivc.mp.	21	
6	Optaflu.mp.	82	
7	("cell-based" adj5 influen* adj5 vaccin*).mp.	87	

#	Searches	Results	Comment
8	(cell adj 5 based adj5 influen* adj5 vaccin*).mp.	0	
9	("Cell-Culture*" adj5 Influenz* adj5 vaccin*).ti,ab,kw.	192	
10	("Cell adj5 Culture*" adj5 Influenz* adj5 vaccin*).ti,ab,kw.	0	
11	or/1-8	875	Base clinical set 1 - Cell-based influenza vaccine terms
12	mdck cell line/ or mdck-i cell line/ or exp mdck-ii cell line/	3751	
13	(mdck or (Madin adj2 Darby)).mp.	13484	
14	(mammalian adj5 cell*).ti,ab,kw.	83500	
15	or/12-14	96463	MDCK or mammalian cell terms
16	influenza vaccine/	39676	
17	influenza a/ or exp "influenza a (h1n1)"/ or exp "influenza a (h3n2)"/	20631	
18	influenza b/	3756	
19	influenz*.mp.	191407	
20	"B/Yamagata lineage".ti,ab,kw.	101	
21	"B/Victoria lineage".ti,ab,kw.	120	
22	or/16-21	191407	Influenza terms
23	15 and 22	4026	Base clinical set 2 - MDCK/mammalian cell influenza vaccine results
24	11 or 23	4831	QIVc or MDCK results
25	limit 24 to human	2326	
26	(human* or people or man or men or female*).ti,ab,kw.	6450742	
27	24 and 26	1612	Human results
28	25 or 27	2947	Final results
29	limit 28 to yr="2016 -Current"	1479	Final results - Publication year limit
30	limit 29 to (english or french or italian or spanish)	1435	Final results - language limit
31	remove duplicates from 30	1420	Duplicates removed

Cochrane

Comment: Includes a number of trials registry entries

ID	Search	Hits	Comment
#1	celtura or "m8-59" or "M8 59"	0	
#2	flucelvax	15	
#3	(quadrivalent NEAR/5 (influen* or flu) NEAR/5 vaccin*)	374	
#4	qivc	10	
#5	iivc	1	

ID	Search	Hits	Comment
#6	Optaflu	10	
#7	("cell-based" NEAR/5 (influen* or flu) NEAR/5 vaccin*)	15	
#8	(cell NEAR/5 based NEAR/5 influen* NEAR/5 vaccin*)	33	
#9	("Cell-Culture*" NEAR/5 (Influenz* or flu) NEAR/5 vaccin*)	80	
#10	(Cell NEAR/5 Culture* NEAR/5 (Influenz* or flu) NEAR/5 vaccin*)	85	
#11	("Cell NEAR/5 Culture*" NEAR/5 Influenz* NEAR/5 vaccin*)	0	
#12	6 to 11	462	Base clinical set 1 - Cell-based influenza vaccine terms
#13	MeSH descriptor: [Madin Darby Canine Kidney Cells] this term only	8	
#14	(mdck or (Madin NEAR/2 Darby))	46	
#15	(mammalian NEAR/5 cell*)	163	
#16	# 6 to #15	204	MDCK and vaccine terms
#17	MeSH descriptor: [Influenza Vaccines] this term only	1621	
#18	MeSH descriptor: [Influenza, Human] this term only	2863	
#19	MeSH descriptor: [Influenza A virus] this term only	408	
#20	MeSH descriptor: [Influenza A Virus, H1N1 Subtype] this term only	383	
#21	MeSH descriptor: [Influenza A Virus, H3N2 Subtype] this term only	226	
#22	MeSH descriptor: [Influenzavirus B] this term only	7	
#23	MeSH descriptor: [Influenza B virus] this term only	296	
#24	influenz*	11041	
#25	"B/Yamagata lineage"	16	
#26	"B/Victoria lineage"	19	
#27	#6 to #26	11041	Influenza terms
#28	#16 and #27	64	Base clinical set 2 -mammalian cell based vaccine results
#29	#12 or #28	497	Final results
#30	#12 or #28 with Cochrane Library publication date Between Jan 2016 and Feb 2022	440	Final results publication year limit

Scopus
TITLE-ABS (celtura OR "m8-59" OR "M8 59" OR flucelvax OR (quadrivalent W/ (influen* OR flu) W/ vaccin*) OR qivc OR iivc OR optaflu OR ("cell-based" W/ vaccin*) OR ("cell based" W/ influenza W/ vaccin*) OR ("cell-culture" W/ influenza W/ vaccin*) OR (cell W/ culture W/ influenz* W/ vaccin*) OR ("Cell W/5 Culture*" W/5 influenz* W/5 vaccin*)) AND (TITLE-ABS (human* OR people OR man OR men OR woman OR women OR male* OR female*)) AND (LIMIT-TO (PUBYEAR , 2022) OR LIMIT-TO (PUBYEAR , 2020) OR LIMIT-TO (PUBYEAR , 2019) OR LIMIT-TO (PUBYEAR , 2018) OR LIMIT-TO (PUBYEAR , 2017) OR LIMIT-TO (PUBYEAR , 2016))

OR

TITLE-ABS(mdck OR (madin W/ darby) OR (mammalian W/ cell*)) AND TITLE-ABS(influen* OR "B/Yamagata lineage" OR "B/Victoria lineage") AND TITLE-ABS(human* OR people OR man OR men OR woman OR women OR male* OR female*) AND (LIMIT-TO(PUBYEAR, 2020) OR LIMIT-TO(PUBYEAR, 2019) OR LIMIT-TO(PUBYEAR, 2018) OR LIMIT-TO(PUBYEAR, 2017) OR LIMIT-TO(PUBYEAR, 2016))
480 refs

Web of Science
SCI-EXPANDED, SSCI, A&HCI, CPCI-S, CPCI-SSH, BKCI-S, BKCI-SSH, ESCI
Timespan=2016-2022

**Set**	**Search**	**Hits**	**Comment**
#1	((ALL=(celtura)) OR ALL=(m8 59)) OR ALL=(flucelvax) OR TI=(quadrivalent NEAR (influen* OR flu) NEAR vaccin*) OR AB=(quadrivalent NEAR (influen* OR flu) NEAR vaccin*) or TS=(quadrivalent NEAR (influen* OR flu) NEAR vaccin*) OR ALL=qivc OR ALL=iivc OR All=Optiflu OR TI=("cell-based" NEAR (influen* or flu) NEAR vaccin*) OR AB=("cell-based" NEAR (influen* or flu) NEAR vaccin*) OR TS=("cell-based" NEAR (influen* or flu) NEAR vaccin*) OR TI=(cell NEAR based NEAR (influen* OR flu) NEAR vaccin*) OR AB=(cell NEAR based NEAR (influen* OR flu) NEAR vaccin*) OR TS=(cell NEAR based NEAR (influen* or flu) NEAR vaccin*) OR TI=("Cell Culture*" NEAR (Influenz* OR flu) NEAR vaccin*) OR AB=("Cell Culture*" NEAR (Influenz* OR flu) NEAR vaccin*) OR TS=("Cell Culture*" NEAR (Influenz* OR flu) NEAR vaccin*)	1,158	Base clinical set 1 - Cell-based influenza vaccine terms
#2	TI=(mdck OR (Madin NEAR Darby)) OR AB=(mdck OR (Madin NEAR Darby)) OR TS=(mdck OR (Madin NEAR Darby)) OR	146,668	MDCK and vaccine terms

Set	Search	Hits	Comment
	TI=(mammalian NEAR cell*) OR AB=(mammalian NEAR cell*) OR TS=(mammalian NEAR cell*)		
#3	TI=(influenz* or "B/Yamagata lineage" OR "B/Victoria lineage") OR AB=(influenz* or "B/Yamagata lineage" OR "B/Victoria lineage") OR TS=(influenz* or "B/Yamagata lineage" OR "B/Victoria lineage")	154,204	Influenza terms
#4	#2 AND #3	3,203	Base clinical set 2 - mammalian cell based vaccine results
#5	#4 OR #1	4,185	Combined results
#6	TI=(human* OR people OR man OR men OR woman OR women OR male* OR female* OR adult* or adolescen* or teen* or geriatric* or child* or infan*) OR AB=(human* OR people OR man OR men OR woman OR women OR male* OR female* OR adult* or adolescen* or teen* or geriatric* or child* or infan*) OR TS=(human* OR people OR man OR men OR woman OR women OR male* OR female* OR adult* or adolescen* or teen* or geriatric* or child* or infan*)	12,322,306	Human textwords
#7	#5 AND #6	1,948	Final results
#8	#5 AND #6 and **2022 or 2020 or 2021 or 2018 or 2017 or 2016 or 2019** (Publication Years)	820	Publication year limit
#9	#5 AND #6 and **2022** or **2020** or **2021** or **2018** or **2017** or **2016** or **2019** (Publication Years) and **English** or **French** or **Italian** or **Spanish** (Languages)	811	Publication language limit

Dissertation Abstracts/Proquest
Search terms/syntax – no articles in Italian
(celtura OR "m8 59" OR flucelvax OR qivc OR iivc OR Optiflu) OR ((quadrivalent NEAR/5 (influen* OR flu) NEAR/5 vaccin*)) OR ti(("cell-based" NEAR/5 (influen* OR flu) NEAR/5 vaccin*)) OR ((cell NEAR/4 based NEAR/5 (influen* OR flu) NEAR/5 vaccin*)) OR ((("cell culture" OR "cell cultures") NEAR/5 (Influenz* OR flu) NEAR/5 vaccin*)) OR ((cell NEAR/2 derived NEAR/5 vaccine*)) OR ((quadrivalent NEAR/2 influenza* NEAR/2 vaccin*) AND ("cell-based" OR "mammalian cell" OR MDCK OR "maudin darby")) = 447 references

Conference Abstracts
Individual Conferences webpages were searched in Google to find links abstracts, posters and program session listings. Programs/presentations/posters were saved in the EndNote library.

- Canadian Immunization Conference
- ECCMID
- ECCMIS-ESCMID elibrary
- ID Week

- Association of Medical Microbiology and Infectious Disease Canada - AMMI Annual Conference
- IPAC Canada
- Global Congress on Infectious Diseases and Clinical Microbiology
- International Meeting on Respiratory Pathogens (ISIRV) and Orthomyxovirus Research Conference part of ESWI
- European Flu Summit
- Annual Meeting on Influenza: Flu & rare diseases
- International Congress on Infectious diseases
- National Immunization Conferences - CDC
- Options X
- Conference series.com Vaccines Conferences – OMICS publishing
- 32nd Annual Congress on immunology and vaccinations scheduled in Vancouver October 30-31, 2020
- Global Summit and expo on vaccines and immunology
- Validate Network - International Conference on vaccines and immunization
- World Vaccine Conference
- Euro Global Summit and Expo on vaccines & vaccination

Meta-search Engines
a) Microsoft Academic Search: https://academic.research.microsoft.com/
All searches were run and results downloaded into EndNote on February 23, 2022
1) Celtura 2016 to 2021 = 0 results
2) Flucelvax 2016 to 2021 = 30 results
3) Qivc 2016 to present = 20 results
4) Optaflu 2016 to present = 8 refs
5) Quadrivalent influenza vaccin* 2016 to present = 632 results – language limits
6) "cell-based" influenza vaccin* 2016 to present = 365 results – language limits
7) "mammalian cell based" AND “influenza vaccin*” 2016 to present = 29 results – language limits
8) "MDCK influenza vaccine" 2016 to present = 4 results Madin Darby influenza vaccin* AND human 2016 to present = 69 results – language limits

b) Science.gov: https://www.science.gov/
All searches were run and results downloaded into EndNote on February 23, 2022
1) Celtura= 0 results
2) Flucelvax 2016 to present= 36 results
3) Qivc 2016 to present = 9 results
4) Optiflu=0 results
5) Quadrivalent influenza vaccin* 2016 to present = 212 results (screen shot below)
6) "cell-based" influenza vaccin* 2016 to present = 171 results
7) "mammalian cell based" AND “influenza vaccine” 2016 to present = 36 results
8) MDCK influenza vaccin* 2016 to present = 93 results
9) Madin Darby influenza vaccin* 2016 to present = 157 results

c) Open Grey - Archived
Closed Summer of 2021 – archived access at https://doi.org/10.17026/dans-xtf-47w5.

1) Quadrivalent influenza vaccin*=0
2) Qivc = 0
3) "cell-based" influenza vaccin* = 0
4) "mammalian cell based" AND "influenza vaccin*" = 0
5) MDCK influenza vaccin* = 0

60 Madin Darby influenza vaccin* = 0

d) Google Scholar

Government sites

- Health Canada
- Public Health Services Canada
- Adverse Events reports
- CDC-United States
- WHO (World Health Organization)
- European Union
- European Centre for Disease Prevention and Control
- European Network or Centres for Pharmacoepidemiology and Pharmacovigilance
- ICAR2021 Conference Portal

Sentinel articles

MEDLINE

34	Izurieta H*.au. and "Relative Effectiveness".ti. and "Journal of Infectious Diseases".jn. and "1255".pg.	1
35	Klein N*.au. and Flucelvax.ti. [****Conference abstract - indexed in EMBASE****]	0
36	Frey S*.au. and "Cell Culture Derived".ti. and "Clinical Infectious Diseases".jn. and "997".pg.	1
37	Perez Rubio A*.au. and "1874".pg. and "Human vaccines & immunotherapeutics".jn.	1
38	barr i*.au. and "cell-culture-derived".ti. and "npj vaccines".jn. [****Epub as of Feb 15, 2022 - indexed in EMBASE****]	0
39	DeMarcus L*.au. and Vaccine.jn. and "4015".pg.	1
40	Bruxvoort K*.au. and Vaccine.jn. and "5807".pg.	1
41	or/34-40 [***Sentinel references****]	5
42	32 and 41 [****Sentinel article verification****]	4
43	41 not 42 [****Missing sentinel article Frey published in 2010****]	1

EMBASE

31	Izurieta H*.au. and "Relative Effectiveness".ti. and "Journal of Infectious Diseases".jn. and "1255".pg.	1
32	Klein N*.au. and Flucelvax.ti.	1
33	Frey S*.au. and "Cell Culture Derived".ti. and "Clinical Infectious Diseases".jn. and "997".pg.	1
34	rubio a*.au. and "1874".pg. and "Human vaccines and immunotherapeutics".jn.	1
35	barr i*.au. and "cell-culture-derived".ti. and "npj vaccines".jn.	1
36	or/31-35 [***Sentinel references****]	5

37	28 and 36 [****Sentinel reference verification****]	4

TABLE S1. Studies assessed for eligibility

	Author, year	Type of record	Reason excluded	Included
1	Boikos, 2018 [1]	Conference poster	Same information as Boikos 2020 paper	
2	Boikos, 2020 [2]	Peer-reviewed publication		Full text
3	Boikos, 2021 (HR) [3]	Peer-reviewed publication		Full text
4	Boikos, 2021 [4]	Peer-reviewed publication		Full text
5	Boikos, 2020 [5]	Conference abstract and poster	Same information as Boikos 2021 paper	
6	Boikos, 2020 [6]	Conference abstract and poster	Same information as Boikos 2021 paper	
7	Bruxvoort, 2019 [7]	Peer-reviewed publication		Full text
8	DeMarcus, 2019 [8]	Peer-reviewed publication		Full text, adults only
9	Divino, 2020 [9]	Peer-reviewed publication		Full text
10	Divino, 2022 [10]	Peer-reviewed publication		Full text
11	Eick-Cost, 2018 [11]	Conference poster		Poster
12	Imran, 2021 (pediatric population) [12]	Conference poster and abstract		Abstract and poster[a]
13	Imran, 2021a (adults) [13]	Conference poster and abstract		Abstract and poster[a]
14	Izurieta, 2019 [14]	Peer-reviewed paper		Full text
15	Izurieta, 2019	Conference poster and abstract	Same information as Izurieta 2019 paper	
16	Izurieta, 2020 [15]	Peer-reviewed publication		Full text
17	Izurieta, 2021 [16]	Peer-reviewed publication		Full text
18	Klein, 2018	Abstract	Same information as Klein 2020 paper	
19	Klein, 2020 [17]	Peer-reviewed publication		Full text
20	Krishnarajah, 2021 [18]	Peer-reviewed publication		Full text
21	Levin, 2021 [19]	Slide deck	Same population as Divino, 2022 paper	
22	Divino, 2021	Conference abstract	Same population as Divino, 2022 paper	
23	Martin, 2019	Conference abstract and poster	Same information as Martin, 2021 paper	
24	Martin, 2021 [20]	Peer-reviewed publication		Full text
25	Pelton, 2020	Conference abstract	Same information as Divino paper	
26	Pelton, 2020	Conference abstract and slide deck	Same information as Krishnarajah, 2021 paper	
27	Pelton, 2021	Conference abstract and poster	Same information as Divino, 2022 paper	
28	Pelton, 2021	Conference poster	Same information as Divino, 2022 paper	
29	Postma, 2020	Conference abstract and poster	Same information as Krishnarajah	

	Author, year	Type of record	Reason excluded	Included
30	Tseng, 2019 [21]	Conference abstract and slide deck		Abstract and slide deck
31	Stuurman, 2020	Report	See #31	
32	Stuurman, 2021 [22]	Peer-reviewed publication	Meta-analysis	
33	Public Health England [23]	Report		Full text

Abbreviations: HR, high risk; peds, pediatrics.

[a] Only available as congress abstracts at the time of the meta-analysis but have since been published in the peer-reviewed literature [24,25].

TABLE S2. Summary of the available aVE and/or rVE data for QIVc, by season, age, influenza definition, vaccine comparator, and population

Study	Season	Age group(s)	Subgroup(s)	Influenza definition	Comparator(s)	Outcome setting	Effect measure[a]	Number (QIVc)	Number (TIV/QIV)	Number (no vaccine)
Boikos, 2020 (cohort; article) [2]	2017–2018	• ≥4<br>• 4 to 17<br>• 18 to 64<br>• ≥65		Clinical diagnosis (code set B)	• QIVe	• Outpatient	OR (MV regression with/without PSM)	92187<br>7465<br>55014<br>29618	1261675<br>404510<br>693014<br>164151	
Boikos, 2021 (cohort; article) [3]	2018–2019	• ≥4 (high risk)	• Any comorbidity<br>• Specific comorbidities[b]	Clinical diagnosis (code set B)	• QIVe	• Any medical encounter	OR (IPTW & DR IPTW)	471301 [e]	1641915 [e]	
Boikos, 2021 (cohort; article) [4]	2018–2019	• ≥4<br>• 4 to 17<br>• 18 to 64<br>• 18 to 49<br>• 50 to 64<br>• ≥65	• Full season<br>• Peak season	Clinical diagnosis (code set B)	• QIVe	• Any medical encounter	OR (IPTW & DR IPTW)	2125430<br>78602<br>700729<br>1529189<br>828460<br>517639	8000903<br>1628038<br>2641268<br>5384922<br>2743654<br>987943	
Bruxvoort, 2019 (TND; article) [7]	2017–2018	• ≥4<br>• 4 to 64<br>• ≥65		Laboratory confirmed:<br>- Any<br>- Influenza A<br>- A(H3N2)<br>- Influenza B	• QIVe/TIVe<br>• No vaccination	• Hospital admission	OR (MV regression)	232<br>75<br>157	5239<br>1741<br>3498	2661<br>1327<br>1334
DeMarcus,[c] 2019 (TND; article) [8]	2017–2018	• ≥18		Laboratory confirmed:<br>- Any<br>- Influenza A<br>- A(H3N2)<br>- A(H1N1)<br>- Influenza B	• TIVe, QIVe, & aTIVe[d]<br>• No vaccination	• Outpatient	OR (MV regression)	506	1002	2529

Study	Season	Age group(s)	Subgroup(s)	Influenza definition	Comparator(s)	Outcome setting	Effect measure[a]	Number (QIVc)	Number (TIV/QIV)	Number (no vaccine)
Divino, 2020 (cohort; article) [9]	2017–2018	• 4 to 64<br>• 4 to 17<br>• 18 to 64<br>• 50 to 64	• Normal risk<br>• High clinical risk^	- All cause hospitalization<br>- Clinical diagnosis (code set B)	• QIVe	• ED/hospital admission<br>• Hospital admission	RR (IPTW)	555538<br>55554<br>499984<br>245548<br>119060 [e]	2528524<br>799014<br>1732039<br>831884<br>491476 [e]	
Divino, 2022 (cohort; article) [10]	2019–2020	• 4 to 64	• Normal risk<br>• High clinical risk^<br>• Full season<br>• Peak season<br>• Shorter season (COVID cut off)	- All cause hospitalization<br>- Clinical diagnosis (code set B)	• QIVe	• ED/hospital admission<br>• Hospital admission	RR (IPTW)	1138969<br>247172[e]	3926357<br>767973[e]	
Eick-Cost, 2018 (TND and cohort, poster) [11]	2017–2018	• 18 to ≥40		- Laboratory confirmed: any influenza<br>- Clinical diagnosis (code set B or ILI)	• QIVe/TIVe	• TND: hospital admission<br>• Cohort:<br>• Outpatient<br>• Hospital admission	TND: OR<br><br>Cohort: IRR (MV regression)	2467<br><br>371394	3239<br><br>3692116	NA
Imran, 2021 (cohort, abstract and poster) [12]	2019–2020	• 4 to 17	• Primary: COVID-19 cut off (March 7/20)<br>• Full season<br>• Peak season<br>• Pre-COVID-19 cut off	Clinical diagnosis (code set B)<br>- Admitting diagnosis<br>- Any diagnosis	• QIVe	• Any medical encounter<br>• Outpatient<br>• Hospital admission or ED visit	OR (DR IPTW)	60480	1240990	NA

Study	Season	Age group(s)	Subgroup(s)	Influenza definition	Comparator(s)	Outcome setting	Effect measure[a]	Number (QIVc)	Number (TIV/QIV)	Number (no vaccine)
Imran, 2021 (cohort, abstract and poster) [13]	2019–2020	• ≥18<br>• 18 to 49<br>• 18 to 64<br>• 50 to 64<br>• ≥65	• Primary: COVID-19 cut off (March 7/20)<br>• Full season<br>• Peak season<br>• Pre-COVID-19 cut off	Clinical diagnosis (code set B)<br>- Admitting diagnosis<br>- Any diagnosis	• QIVe	• Any medical encounter<br>• Outpatient<br>• ED/hospital admission	OR (DR IPTW)	1499215<br>533073<br>1144427<br>611354<br>533073	4126263<br>1726866<br>3427818<br>1700952<br>1726866	NA
Izurieta, 2019 (cohort; article) [14]	2017–2018	• ≥65	• Full season<br>• Limited season	- Clinical diagnosis (code set B & J129)<br>- Rapid antigen test & oseltamivir prescription	• QIVe<br>• TIVe<br>Not in scope:<br>• aTIVe<br>• HD TIVe	• ED/hospital admission<br>• Outpatient<br>• Hospital admission	RR (IPTW & DR IPTW)	659,249	1863654<br>1018494	NA
Izurieta, 2020 (cohort; article) [15]	2018–2019	• ≥65		Clinical diagnosis (code set B & J129)	• QIVe<br>Not in scope:<br>• aTIV<br>• HD TIVe	• ED/hospital admission<br>• Hospital admission	RR (IPTW & DR IPTW)	761037	1454340	
Izurieta, 2021 (cohort; article) [16]	2019–2020	• ≥65	• Region-weeks with ≥15% positive respiratory sample<br>• Flu circulation based on Medicare antiviral	Clinical diagnosis (code set B & J129)	• QIVe<br>Not in scope:<br>• aTIVe<br>• HD TIVe	• ED/hospital admission<br>• Hospital admission	RR (IPTW & DR IPTW)	813838	1573548	

Study	Season	Age group(s)	Subgroup(s)	Influenza definition	Comparator(s)	Outcome setting	Effect measure[a]	Number (QIVc)	Number (TIV/QIV)	Number (no vaccine)
			prescription rates							
Klein, 2020 (TND & cohort; article) [17]	2017–2018	• 4 to 64<br><br><br>• 4 to 64<br>• 4 to 17<br>• 18 to 64		Laboratory confirmed:<br>- Influenza A<br>- Influenza B	• QIVe/TIVe (A)<br>• TIVe (B)<br>• No vaccination	• Any medical encounter	TND: OR<br><br><br>Cohort: HR (MV regression)	925<br><br><br>84420<br>43735<br>40685	10007<br><br><br>932545<br>220419<br>712126	17473<br><br><br>2036283<br>380305<br>1677978
Krishnarajah, 2021 (cohort; article) [18]	2018–2019	• 4 to 64<br>• 4 to 17<br>• 18 to 64	• Average risk<br>• High clinical risk[e]<br>• Full season<br>• Peak season	- All cause hospitalization<br>- Clinical diagnosis (code set B)	• QIVe	• ED/hospital admission<br>• Hospital admission	RR (IPTW)	665047<br>74304<br>590705<br><br>142150[e]	3062843<br>839744<br>2223435<br><br>584959[e]	
Martin, 2021 (TND; article) [20]	2017–2018	• ≥18		Laboratory confirmed:<br>- Any<br>- A(H3N2)<br>- B/Yamagata	• QIVe/TIVe<br>• QIVe/TIVe/ HD TIV/aTIV in ≥65 years<br>• No vaccination	• Hospital admission	OR	65	1676	1174
Public Health England, 2020 (TND; report) [23]	2019–2020	• 18 to 64<br>• ≥65		Laboratory confirmed:<br>- Any<br>- A(H3N2)	• QIVe & aTIV<br>• No vaccination	• Outpatient (primary care)	OR	Not reported	Not reported	Not reported
Tseng, 2019 (TND; abstract & slide deck) [21]	2018–2019	• ≥4<br>• 4 to 64<br>• ≥65		Laboratory confirmed:<br>- Any<br>- Influenza A<br>- A(H3N2)	• QIVe/TIVe<br>• No vaccination	• Hospital admission	OR	1047<br>351<br>696	4156<br>1383<br>2773	1979<br>1008<br>971

Study	Season	Age group(s)	Subgroup(s)	Influenza definition	Comparator(s)	Outcome setting	Effect measure[a]	Number (QIVc)	Number (TIV/QIV)	Number (no vaccine)
				- A(H1N1)<br>- A (not typed)						

Abbreviations and definitions: any medical encounter, includes outpatient visits, hospital admissions and emergency department visits; aTIV, adjuvanted egg-based trivalent influenza vaccine; DR IPTW, doubly robust IPTW; ED/hospital admission, includes both hospital admissions and emergency department visits; HD TIV, high dose egg-based trivalent influenza vaccine; HR, hazard ratio; IPTW, inverse probability of treatment weighting; IRR, incidence rate ratio; MV, multivariable; OR, odds ratio; PSM, propensity score matched; QIVe, egg-based quadrivalent influenza vaccine; RR, relative risk: TIVe, egg-based trivalent influenza vaccine; QIVe/TIVe, both vaccines were used by participants/database members; TND, test negative design.

[a] Including statistical adjustments for confounding made to estimates of VE or rVE

[b] Specific comorbidities+: analyses were available for: chronic obstructive pulmonary disease, asthma, myocardial infarction or congestive heart failure, cardiovascular disease or peripheral vascular disease, renal disease, diabetes, any malignancy, HIV/AIDS, rheumatic disease, and liver disease but are not presented in this review.

[c] The results for ≥6 months to 17 years are not presented because the comparison group included children ineligible for vaccination with QIVc for the seasons under study.

[d] 0.2% of TIVe recipients received aTIVe

[e] High clinical risk: individuals in this group had chronic heart disease, kidney disease, liver disease, neurological disease, and/or respiratory disease, or diabetes, immunosuppression, morbid obesity, asplenia or dysfunction of the spleen, and/or were pregnant

TABLE S3. Assessment of risk of bias using ROBINS-I tool for relative vaccine effectiveness estimates (N=18)

Study	Design, Description	Outcome	Risk of confounding	Risk of selection bias	Risk of mis-classification of exposure	Risk of deviation from intended vaccine	Risk of missing data	Risk of mis-classification of outcomes	Risk of reporting bias	Overall risk of bias
Bruxvoort, 2019 [7]	Test-negative	aVE & rVE	Moderate	Moderate	Low	Low	Moderate	Moderate	Low	**Moderate**
Tseng, 2019 [21]	Test-negative	aVE & rVE	Moderate	Moderate	Low	Low	NA	Moderate	Low	**Moderate**
Eick-Cost, 2018 [11]	Test-negative	rVE	Moderate	Moderate	Low	Low	Moderate	Moderate	Low	**Moderate**
	Cohort		Moderate	Moderate	Low	Low	Moderate	Moderate	Low	**Moderate**
Public Health England, 2020 [23]	Test-negative	aVE	Serious (lacked detail)	Serious (lacked detail)	Low	Low	Serious (lacked detail)	Serious (lacked detail)	Low	**Serious**
DeMarcus, 2019 [8]	Test-negative, 18+ years	aVE & rVE	Serious	Moderate	Low	Low	Moderate	Moderate	Low	**Moderate**
	Test-negative, 6 months to 17 years		Critical	Moderate	Serious	Low	Serious	Critical	Moderate	**Critical**
Martin, 2021 [20]	Test-negative	aVE & rVE	Moderate	Moderate	Low	Low	Moderate	Moderate	Low	**Moderate**
Klein, 2020 [17]	Test-negative	aVE & rVE	Moderate	Moderate	Low	Low	Moderate	Moderate	Moderate	**Moderate**
	Cohort		Moderate	Moderate	Low	Low	Moderate	Moderate	Moderate	**Moderate**
Izurieta, 2019 [14]	Cohort	rVE	Low	Moderate	Low	Low	Low	Low	Low	**Moderate**
Boikos, 2020 [2]	Cohort, not PSM	rVE	Moderate	Moderate	Low	Low	Moderate	Low	Low	**Moderate**
	Cohort, PSM	rVE	Low	Moderate	Low	Low	Moderate	Low	Low	**Moderate**
Imran, 2021 [12]	Cohort 4-17 years	rVE	Low	Moderate	Low	Low	Moderate	Low	Low	**Moderate**
Imran, 2021 [13]	Cohort 18+ years	rVE	Low	Moderate	Low	Low	Moderate	Low	Low	**Moderate**

Study	Design, Description	Outcome	Risk of confounding	Risk of selection bias	Risk of mis-classification of exposure	Risk of deviation from intended vaccine	Risk of missing data	Risk of mis-classification of outcomes	Risk of reporting bias	Overall risk of bias
Boikos, 2021 [4]	Cohort	rVE	Low	Moderate	Low	Low	Moderate	Low	Low	**Moderate**
Boikos, 2021 [3]	Cohort<br>High risk	rVE	Low	Moderate	Low	Low	Moderate	Low	Low	**Moderate**
Divino, 2020 [9]	Cohort	rVE	Low	Moderate	Low	Low	Low	Low	Low	**Moderate**
Krishnarajah, 2021 [18]	Cohort	rVE	Low	Moderate	Low	Low	Low	Low	Low	**Moderate**
Divino, 2022 [10]	Cohort	rVE	Low	Moderate	Low	Low	Moderate	Low	Low	**Moderate**
Izurieta, 2020 [15]	Cohort	rVE	Low	Moderate	Low	Low	Moderate	Low	Low	**Moderate**
Izurieta, 2021 [16]	Cohort	rVE	Low	Moderate	Low	Low	Moderate	Low	Low	**Moderate**

Abbreviations: aVE, absolute vaccine effectiveness; PSM, propensity score matched; ROBINS-I, risk of bias in non-randomized studies of interventions; rVE, relative vaccine effectiveness.

FIGURE S1. Forest plot of aVE estimates of QIVc and QIVe/TIVe against laboratory-confirmed influenza (any type), 2017–2018 to 2019–2020.

Estimates for QIVe/TIVe are limited to those reported in the included studies for QIVc effectiveness. adm, admission; aVE, adjusted absolute vaccine effectiveness; Hosp, hospital; I^2, heterogeneity measure; Outpt, outpatient; QIVc, cell-based quadrivalent inactivated influenza vaccine; QIVe, egg-based quadrivalent inactivated influenza vaccine; TIVe, egg-based trivalent inactivated influenza vaccine.

Study	Age	Setting/Season	aVE (95% CI)	% Weight
**QIVc**				
Bruxvoort, 2019	≥4	Hosp adm/2017–2018	14 (–31 to 43)	14.55
Martin, 2020	≥18	Hosp adm/2017–2018	40.1 (–15.2 to 68.8)	12.41
DeMarcus, 2019	≥18	Outpt/2017–2018	52 (36–64)	29.78
Tseng, 2019	≥65	Hosp adm/2018–2019	–14 (–84 to 29)	8.1
Tseng, 2019	4–64	Hosp adm/2018–2019	20 (–40 to 54)	10.65
PHE, 2020	≥65	Outpt/2019–2020	31.7 (–81.5 to 74.3)	4.77
PHE, 2020	18–64	Outpt/2019–2020	63.9 (26.6–82.2)	19.75
**Overall (I^2 = 45%)**			**37.6 (19.4–55.9)**	**100**
**TIVe/QIVe**				
Bruxvoort, 2019	≥4	Hosp adm/2017–2018	–2 (–20 to 14)	19.48
Martin, 2020	≥18	Hosp adm/2017–2018	34.5 (21.1–45.7)	20.98
DeMarcus, 2019	≥18	Outpt/2017–2018	51 (35–63)	20.47
Tseng, 2019	≥65	Hosp adm/2018–2019	–10 (–62 to 25)	10.63
Tseng, 2019	4–64	Hosp adm/2018–2019	31 (–4 to 54)	15.14
PHE, 2020	18–64	Outpt/2019–2020	38.9 (–4.5 to 64.3)	13.3
**Overall (I^2 = 81%)**			**26.1 (6.7–45.4)**	**100**

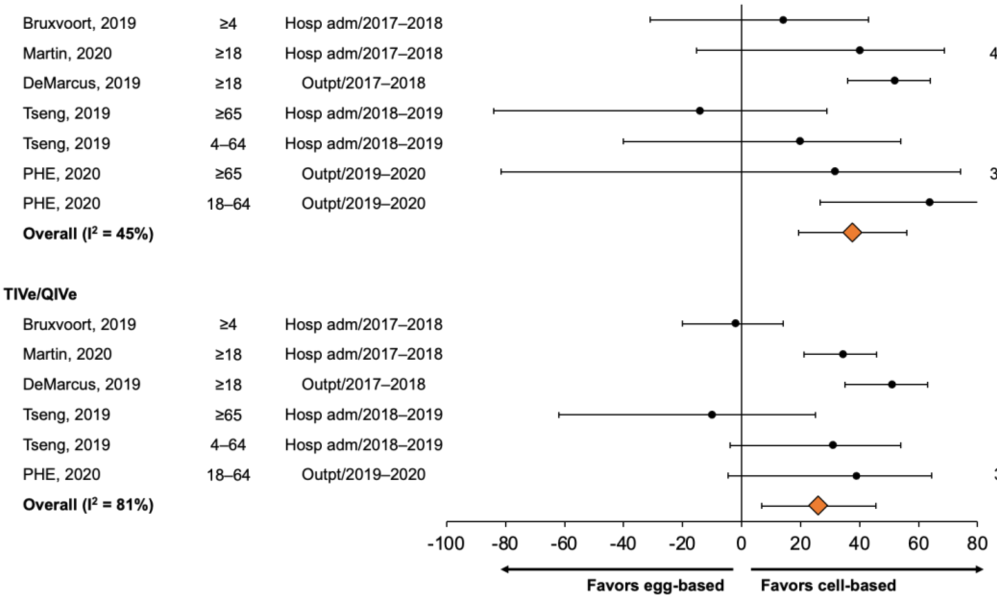

FIGURE S2. rVE estimates of QIVc compared with QIVe (or QIVe/TIVe) in preventing influenza-related medical encounters, 2017–2018 to 2019–2020.
(*a*) Forest plot of overall and by study design. Pooled rVE and heterogeneity estimates were calculated separately for each design and overall; the overall estimate was not stratified by any variable. (*b*) Galbraith plot by study in Panel *a*. adm, admission; CI, 95% confidence interval; ED, emergency department; hosp, hospital; I^2, heterogeneity measure; Outpt, outpatient; QIVc, cell-based quadrivalent inactivated influenza vaccine; QIVe, egg-based quadrivalent inactivated influenza vaccine; rVE, relative vaccine effectiveness; TIVe, egg-based trivalent inactivated influenza vaccine.

a

b

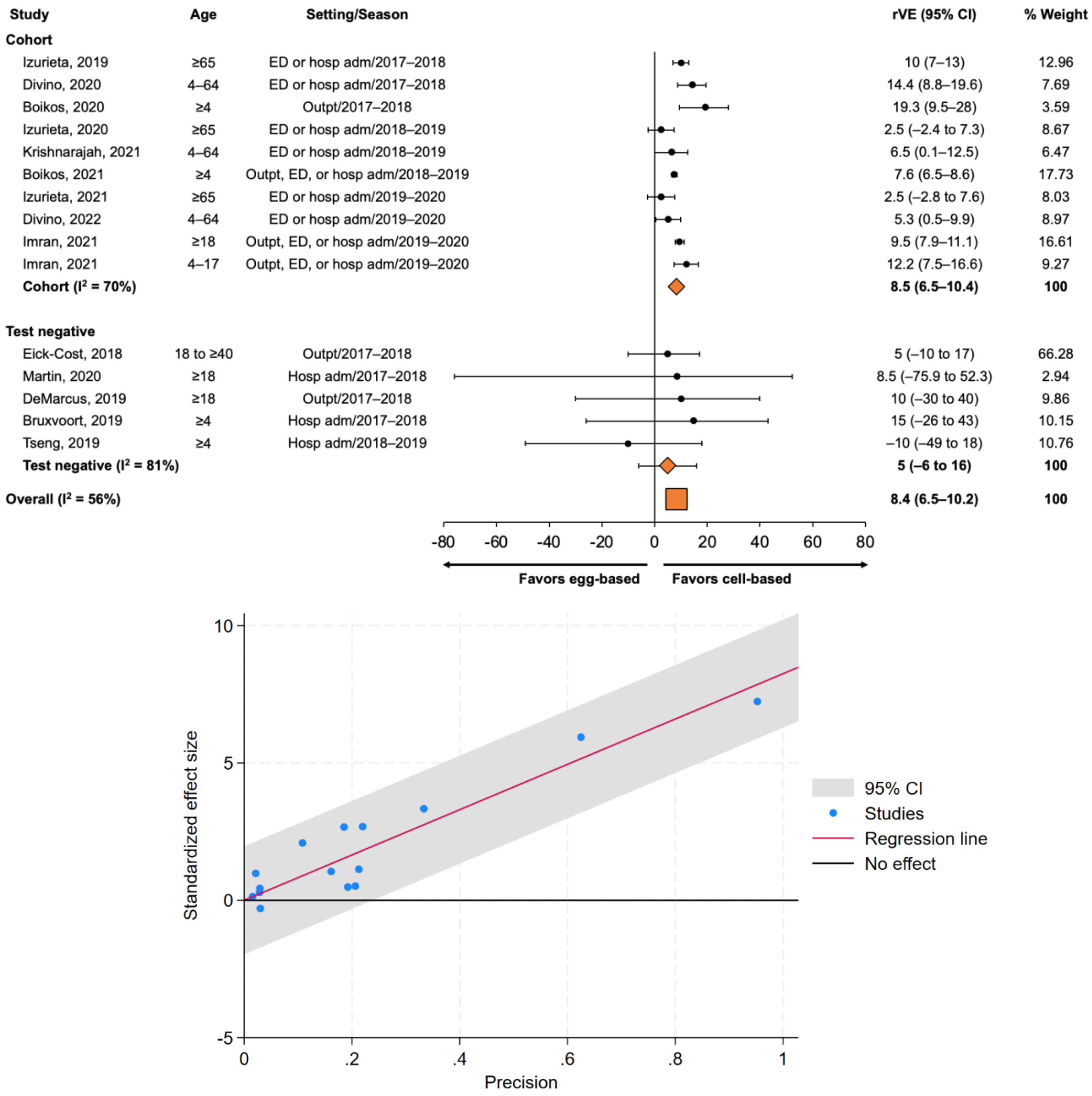

FIGURE S3. Forest plot of rVE estimates of QIVc compared with QIVe (or QIVe/TIVe) against laboratory-confirmed influenza, by influenza type.

(*a*) Any influenza type or subtype, 2017–2018 and 2018–2019 seasons. (*b*) Influenza A, 2017–2018 and 2018–2019 seasons. (*c*) A(H3N2), 2017–2018 and 2018–2019 seasons. adm, admission; ED, emergency department; hosp, hospital; I^2, heterogeneity measure; Outpt, outpatient; QIVc, cell-based quadrivalent inactivated influenza vaccine; QIVe, egg-based quadrivalent inactivated influenza vaccine; rVE, relative vaccine effectiveness; TIVe, egg-based trivalent inactivated influenza vaccine.

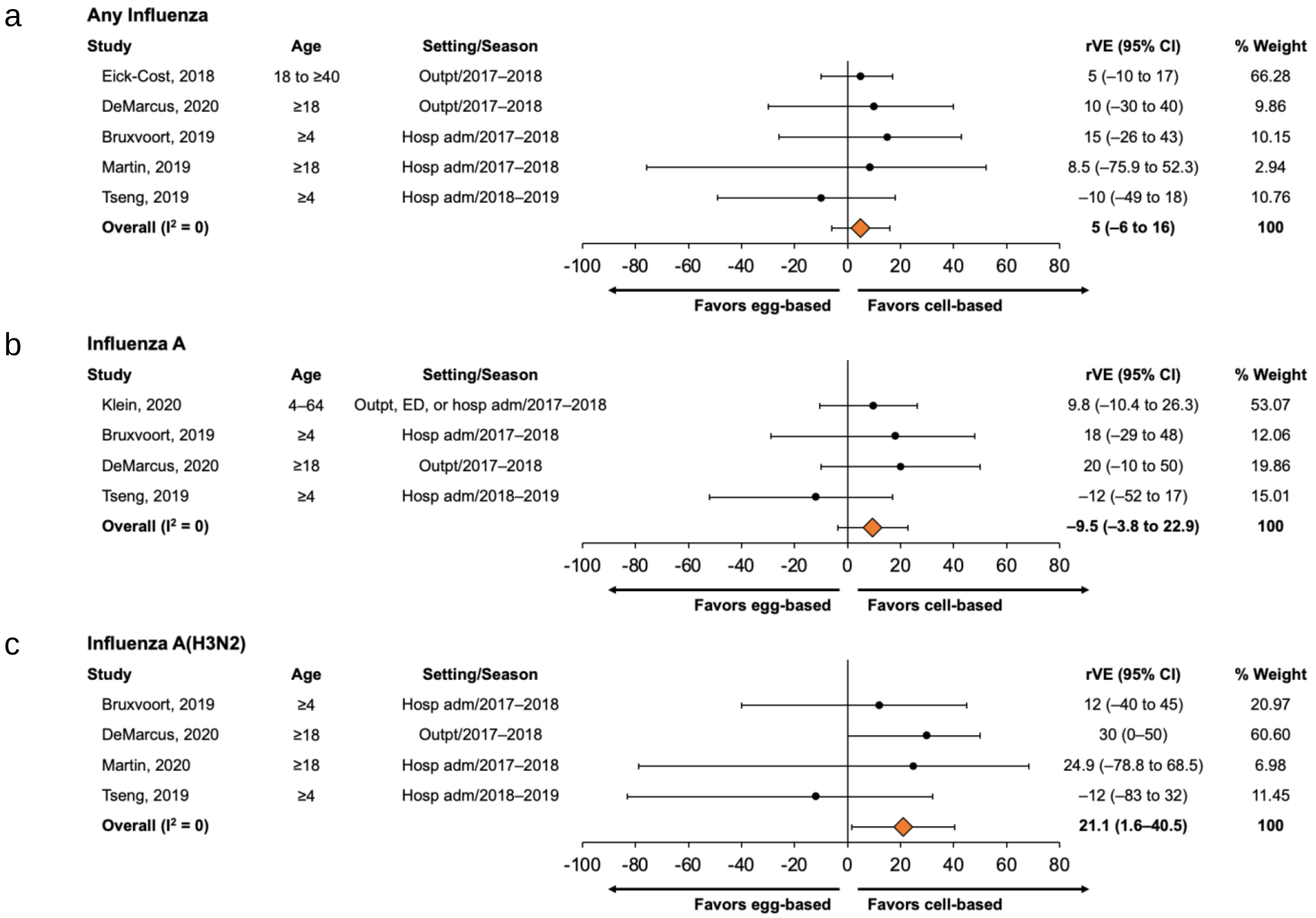

FIGURE S4. Forest plot of rVE estimates of QIVc compared with QIVe (or QIVe/TIVe) in preventing medical encounters by clinical setting, all ages, 2017–2018 to 2019–2020. Pooled rVE estimates were calculated separately for each setting. adm, admission; ED, emergency department; hosp, hospital; I^2, heterogeneity measure; neg, negative; QIVc, cell-based quadrivalent inactivated influenza vaccine; QIVe, egg-based quadrivalent inactivated influenza vaccine; rVE, relative vaccine effectiveness; TIVe, egg-based trivalent inactivated influenza vaccine.

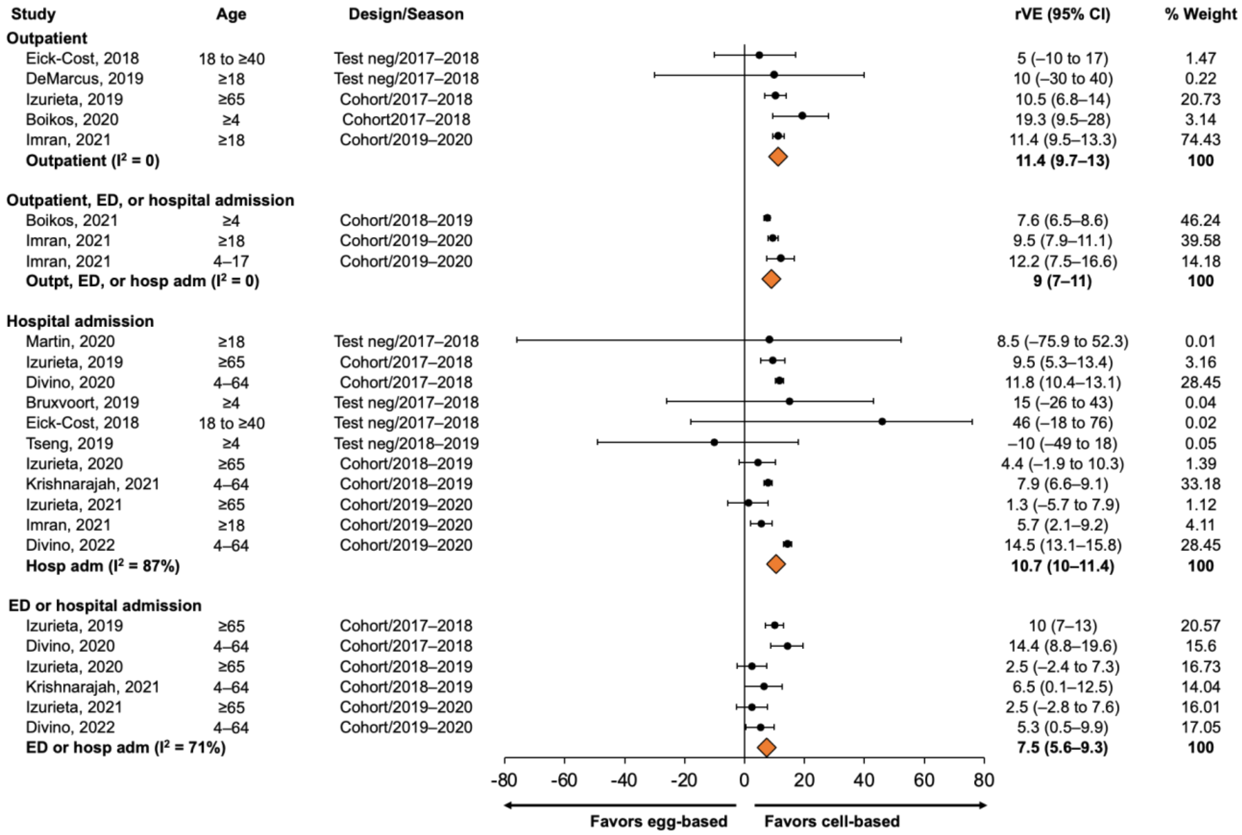

FIGURE S5. Forest plot of rVE estimates of QIVc compared with QIVe (or QIVe/TIVe) in preventing medical encounters, by season, 2017–2018 to 2019–2020.
Pooled rVE estimates were calculated separately for each season; the overall estimate was not stratified by any variable. adm, admission; ED, emergency department; Hosp, hospital; I sq, I^2 (heterogeneity measure); Outpt, outpatient; QIVc, cell-based quadrivalent inactivated influenza vaccine; QIVe, egg-based quadrivalent inactivated influenza vaccine; rVE, relative vaccine effectiveness; TIVe, egg-based trivalent inactivated influenza vaccine.

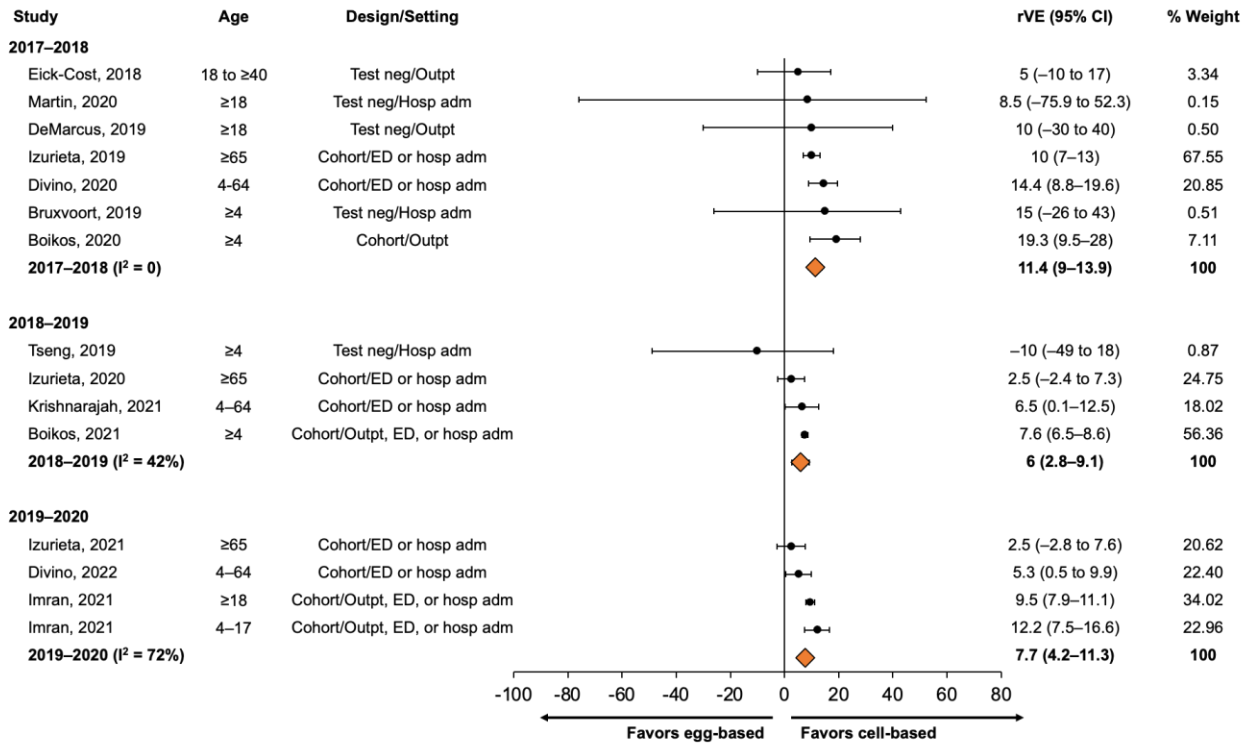

FIGURE S6. Forest plot of rVE estimates of QIVc compared with QIVe (or QIVe/TIVe) in preventing medical encounters by age group, 2017–2018 to 2019–2020.

Pooled rVE estimates were calculated separately for each age group. adm, admission; ED, emergency department; hosp, hospital; I^2, heterogeneity measure; Outpt, outpatient; QIVc, cell-based quadrivalent inactivated influenza vaccine; QIVe, egg-based quadrivalent inactivated influenza vaccine; rVE, relative vaccine effectiveness; TIVe, egg-based trivalent inactivated influenza vaccine. *Study population 18-40 years of age.

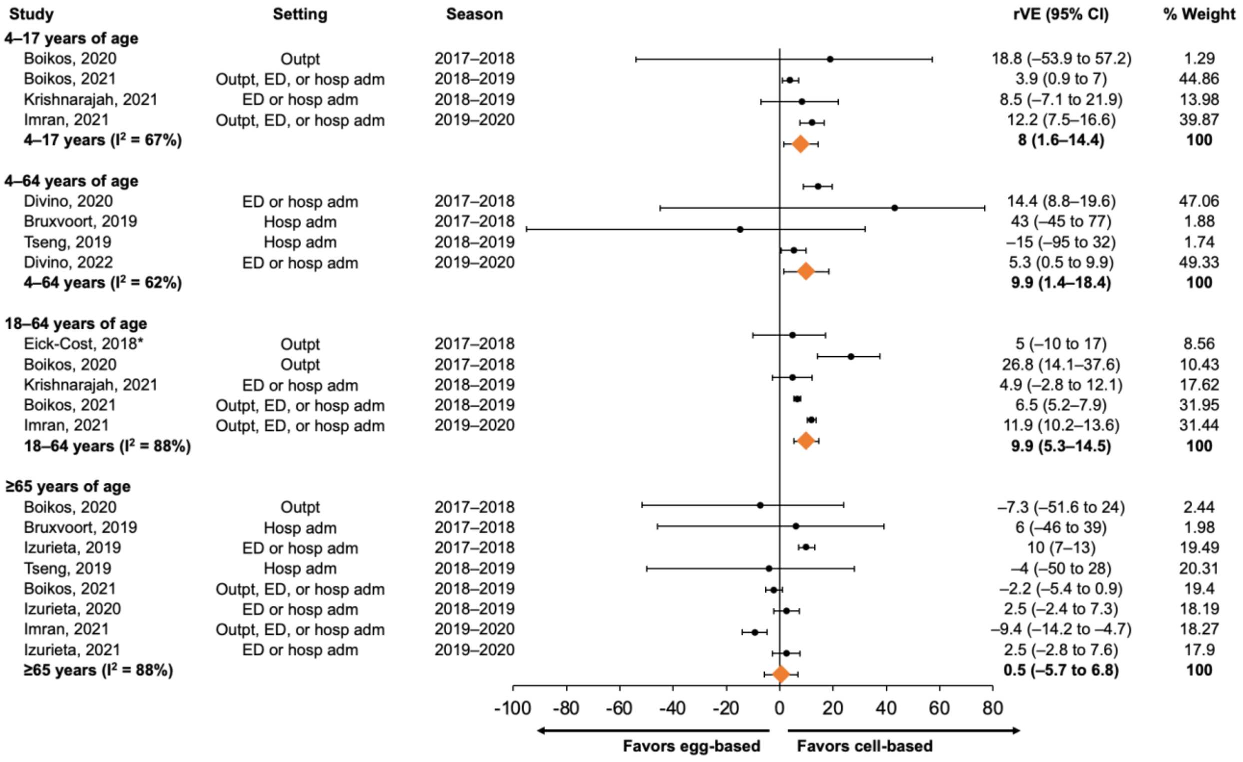

FIGURE S7. Forest plot of rVE estimates of QIVc compared with QIVe in preventing medical encounters for populations at higher risk of serious outcomes compared with the general population, 2017–2018 to 2019–2020.

Pooled rVE estimates were calculated separately for each group. adm, admission; ED, emergency department; hosp, hospital; I^2, heterogeneity measure; Outpt, outpatient; QIVc, cell-based quadrivalent inactivated influenza vaccine; QIVe, egg-based quadrivalent inactivated influenza vaccine; rVE, relative vaccine effectiveness; TIVe, egg-based trivalent inactivated influenza vaccine.

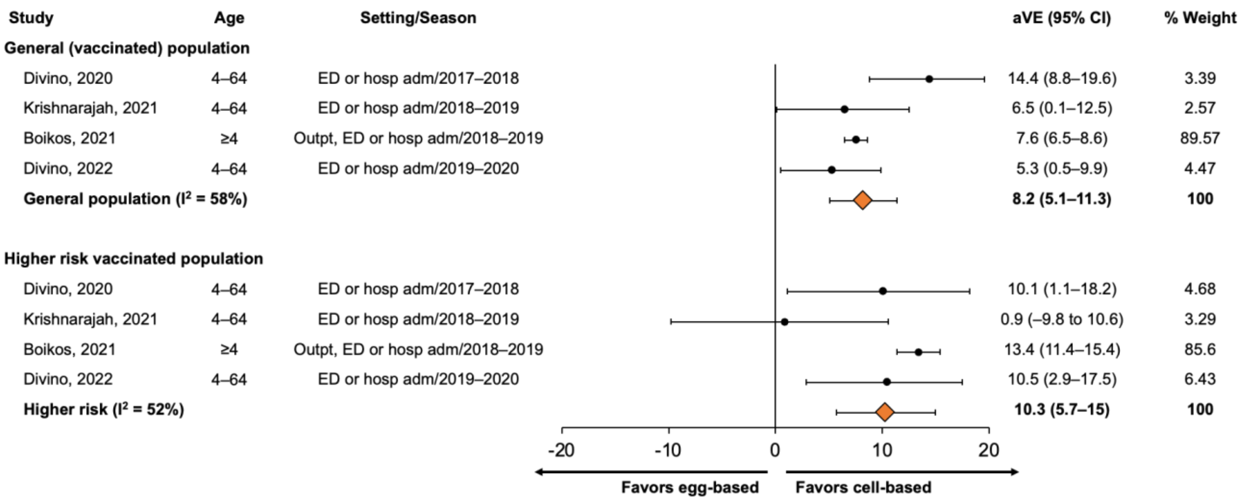

References

1. Boikos, C.; Sylvester, G.; Sampalis, J.; Mansi, J. Effectiveness of the cell culture-based and egg-based, seasonal influenza vaccines during the 2017-2018 Northern Hemisphere influenza season. In Proceedings of the Canadian Immunization Conference, Ottawa, Canada, 2018.
2. Boikos, C.; Sylvester, G.C.; Sampalis, J.S.; Mansi, J.A. Relative effectiveness of the cell-cultured quadrivalent influenza vaccine compared to standard, egg-derived quadrivalent influenza vaccines in preventing influenza-like illness in 2017-2018. *Clinical infectious diseases : an official publication of the Infectious Diseases Society of America* **2020**, *71*, e665-671, doi:10.1093/cid/ciaa371.
3. Boikos, C.; Imran, M.; Nguyen, V.H.; Ducruet, T.; Sylvester, G.C.; Mansi, J.A. Effectiveness of the Cell-Derived Inactivated Quadrivalent Influenza Vaccine in Individuals at High Risk of Influenza Complications in the 2018-2019 United States Influenza Season. *Open forum infectious diseases* **2021**, *8*, ofab167, doi:10.1093/ofid/ofab167.
4. Boikos, C.; Fischer, L.; O'Brien, D.; Vasey, J.; Sylvester, G.C.; Mansi, J.A. Relative Effectiveness of the Cell-derived Inactivated Quadrivalent Influenza Vaccine Versus Egg-derived Inactivated Quadrivalent Influenza Vaccines in Preventing Influenza-related Medical Encounters During the 2018-2019 Influenza Season in the United States. *Clinical infectious diseases : an official publication of the Infectious Diseases Society of America* **2021**, *73*, e692-e698, doi:10.1093/cid/ciaa1944.
5. Boikos, C.; Imran, M.; Nguyen, V.; Ducruet, T.; Sylvester, G.C.; Mansi, J.A. Relative effectiveness of cell-derived versus egg-derived quadrivalent influenza vaccines in individuals with underlying medical conditions in the U.S. 2018-2019 influenza season. In Proceedings of the European Scientific Working group on Influenza (ESWI), Valencia, Spain, 2020.
6. Boikos, C.; Fischer, L.; O'Brien, D.; Vasey, J.; Sylvester, G.C.; Mansi, J.A. Relative effectiveness of cell-derived quadrivalent inactivated influenza vaccine (ccIIV4) versus egg-derived IIV4 in preventing influenza-related medical encounters during the 2018-2019 influenza season in the United States. In Proceedings of the European Scientific Working group on Influenza (ESWI), Valencia, Span, 2020.
7. Bruxvoort, K.J.; Luo, Y.; Ackerson, B.; Tanenbaum, H.C.; Sy, L.S.; Gandhi, A.; Tseng, H.F. Comparison of vaccine effectiveness against influenza hospitalization of cell-based and egg-based influenza vaccines, 2017-2018. *Vaccine* **2019**, *37*, 5807-5811, doi:10.1016/j.vaccine.2019.08.024.
8. DeMarcus, L.; Shoubaki, L.; Federinko, S. Comparing influenza vaccine effectiveness between cell-derived and egg-derived vaccines, 2017-2018 influenza season. *Vaccine* **2019**, *37*, 4015-4021, doi:10.1016/j.vaccine.2019.06.004.
9. Divino, V.; Krishnarajah, G.; Pelton, S.I.; Mould-Quevedo, J.; Anupindi, V.R.; DeKoven, M.; Postma, M.J. A real-world study evaluating the relative vaccine effectiveness of a cell-based quadrivalent influenza vaccine compared to egg-based quadrivalent influenza vaccine in the US during the 2017-18 influenza season. *Vaccine* **2020**, *38*, 6334-6343, doi:10.1016/j.vaccine.2020.07.023.
10. Divino, V.; Ruthwik Anupindi, V.; DeKoven, M.; Mould-Quevedo, J.; Pelton, S.I.; Postma, M.J.; Levin, M.J. A Real-World Clinical and Economic Analysis of Cell-

Derived Quadrivalent Influenza Vaccine Compared to Standard Egg-Derived Quadrivalent Influenza Vaccines During the 2019-2020 Influenza Season in the United States. *Open forum infectious diseases* **2022**, *9*, ofab604, doi:10.1093/ofid/ofab604.
11. Eick-Cost, A.; Hu, Z. Relative effectiveness of cell-based influenza vaccines compared to egg-based influenza vaccines, active component U.S. Service members, 2017-18 season. In Proceedings of the International Conference on Emerging Infectious Diseases, Atlanta, GA, August 26-29, 2018; p. 54. Abstr. 129.
12. Imran, M.; Ortiz, J.R.; McLean, H.Q.; Fisher, L.; O'Brien, D.; Bonafede, M.; Mansi, J.A.; Boikos, C. Relative effectiveness of cell derived quadrivalent influenza vaccine versus egg derived quadrivalent influenza vaccine in preventing influenza related medical encounters in a pediatric population during the 2019-2020 US influenza season. In Proceedings of the International Society for Influenza and other Respiratory Virus Diseases–World Health Organization Virtual Conference: COVID-19, Influenza and RSV: Surveillance-Informed Prevention and Treatment, https://isirv.org/site/index.php/9-events/528-isirv-who-virtual-conf-oct21, 19-21 October 2021, 2021.
13. Imran, M.; Ortiz, J.; McLean, H.; Fisher, L.; O'Brien, D.; Bonafede, M.; al., e. Relative effectiveness of cell–derived quadrivalent influenza vaccine (IIV4c) versus egg-derived quadrivalent influenza vaccine (IIV4e) in preventing influenza-related medical encounters in adults during the 2019-2020 influenza season in the United States. In Proceedings of the International Society for Influenza and other Respiratory Virus Diseases–World Health Organization Virtual Conference: COVID-19, Influenza and RSV: Surveillance-Informed Prevention and Treatment, https://isirv.org/site/index.php/9-events/528-isirv-who-virtual-conf-oct21, 19-21 October 2021, 2021.
14. Izurieta, H.S.; Chillarige, Y.; Kelman, J.; Wei, Y.; Lu, Y.; Xu, W.; Lu, M.; Pratt, D.; Chu, S.; Wernecke, M.; et al. Relative effectiveness of cell-cultured and egg-based influenza vaccines among elderly persons in the United States, 2017-2018. *J Infect Dis* **2019**, *220*, 1255-1264, doi:10.1093/infdis/jiy716.
15. Izurieta, H.S.; Chillarige, Y.; Kelman, J.; Wei, Y.; Lu, Y.; Xu, W.; Lu, M.; Pratt, D.; Wernecke, M.; MaCurdy, T.; et al. Relative Effectiveness of Influenza Vaccines Among the United States Elderly, 2018-2019. *J Infect Dis* **2020**, *222*, 278-287, doi:10.1093/infdis/jiaa080.
16. Izurieta, H.S.; Lu, M.; Kelman, J.; Lu, Y.; Lindaas, A.; Loc, J.; Pratt, D.; Wei, Y.; Chillarige, Y.; Wernecke, M.; et al. Comparative Effectiveness of Influenza Vaccines Among US Medicare Beneficiaries Ages 65 Years and Older During the 2019-2020 Season. *Clinical infectious diseases : an official publication of the Infectious Diseases Society of America* **2021**, *73*, e4251-e4259, doi:10.1093/cid/ciaa1727.
17. Klein, N.P.; Fireman, B.; Goddard, K.; Zerbo, O.; Asher, J.; Zhou, J.; King, J.; Lewis, N. Vaccine effectiveness of cell-culture relative to egg-based inactivated influenza vaccine during the 2017-18 influenza season. *PLoS One* **2020**, *15*, e0229279, doi:10.1371/journal.pone.0229279.
18. Krishnarajah, G.; Divino, V.; Postma, M.J.; Pelton, S.I.; Anupindi, V.R.; DeKoven, M.; Mould-Quevedo, J. Clinical and Economic Outcomes Associated with Cell-Based Quadrivalent Influenza Vaccine vs. Standard-Dose Egg-Based Quadrivalent Influenza Vaccines during the 2018-19 Influenza Season in the United States. *Vaccines (Basel)* **2021**, *9*, doi:10.3390/vaccines9020080.

19. Levin, M.J.; Divino, V.; Anupindi, V.R.; DeKoven, M.; Mould-Quevedo, J.; Pelton, S.I.; et al. Relative vaccine effectiveness against influenza-related and any respiratory-related hospital encounter during the 2019/20 high influenza activity period. a comprehensive real-world analysis to compare quardravalent cell-based and egg-based influenza vaccines. In Proceedings of the IDWeek, Washington, DC, 2021.
20. Martin, E.T.; Cheng, C.; Petrie, J.G.; Alyanak, E.; Gaglani, M.; Middleton, D.B.; Ghamande, S.; Silveira, F.P.; Murthy, K.; Zimmerman, R.K.; et al. Low Influenza Vaccine Effectiveness Against A(H3N2)-Associated Hospitalizations in 2016-2017 and 2017-2018 of the Hospitalized Adult Influenza Vaccine Effectiveness Network (HAIVEN). *J Infect Dis* **2021**, *223*, 2062-2071, doi:10.1093/infdis/jiaa685.
21. Tseng, H.F.; Bruxvoort, K.J.; Luo, Y.; Ackerson, B.; Tanenbaum, H.C.; Sy, L.S. Vaccine effectiveness against influenza hospitalization in the 2018-2019 season: comparison between cell-based and egg-based influenza vaccines [Abstract 10395]. In Proceedings of the Options X for the Control of Influenza Singapore, 2019; p. Abstract 10395.
22. Stuurman, A.L.; Biccler, J.; Carmona, A.; Descamps, A.; Díez-Domingo, J.; Muñoz Quiles, C.; Nohynek, H.; Rizzo, C.; Riera-Montes, M. Brand-specific influenza vaccine effectiveness estimates during 2019/20 season in Europe - Results from the DRIVE EU study platform.
23. Public Health England. Surveillance of influenza and other respiratory viruses in the UK: Winter 2019 to 2020. Available online: https://webarchive.nationalarchives.gov.uk/ukgwa/20220401215804/https://www.gov.uk/government/statistics/annual-flu-reports (accessed on 1 December 2022).
24. Imran, M.; Ortiz, J.R.; McLean, H.Q.; Fisher, L.; O'Brien, D.; Bonafede, M.; Mansi, J.A.; Boikos, C. Relative Effectiveness of Cell-Based Versus Egg-Based Quadrivalent Influenza Vaccines in Adults During the 2019-2020 Influenza Season in the United States. *Open forum infectious diseases* **2022**, *9*, ofac532, doi:10.1093/ofid/ofac532.
25. Imran, M.; Ortiz, J.R.; McLean, H.Q.; Fisher, L.; O'Brien, D.; Bonafede, M.; Mansi, J.A.; Boikos, C. Relative Effectiveness of Cell-based Versus Egg-based Quadrivalent Influenza Vaccines in Children and Adolescents in the United States During the 2019-2020 Influenza Season. *Pediatr Infect Dis J* **2022**, *41*, 769-774, doi:10.1097/INF.0000000000003620.
